# From ABC to KPZ

**DOI:** 10.1007/s00440-024-01314-z

**Published:** 2024-10-22

**Authors:** G. Cannizzaro, P. Gonçalves, R. Misturini, A. Occelli

**Affiliations:** 1 Department of Statistics, University of Warwick, Zeeman Building, Coventry, CV4 7AL UK; 2 Department of Mathematics, Instituto Superior Técnico, Av. Rovisco Pais 1, Lisbon, 1049-001 Portugal; 3 Instituto de Matemática e Estatística, Universidade Federal do Rio Grande do Sul, Av. Bento Gonçalves, 9500, Porto Alegre, CEP 91509-900 Brazil; 4 LAREMA, Université d’Angers, 2 Bd de Lavoisier, 49045 Angers, France

**Keywords:** Stochastic Burgers equation, Ornstein-Uhlenbeck process, KPZ equation, Multi-component, Crossover weakly asymmetric exclusion, Two species, 60K35, 82C22

## Abstract

We study the equilibrium fluctuations of an interacting particle system evolving on the discrete ring with $N\in {\mathbb {N}}$ points, denoted by ${\mathbb {T}}_N$, and with three species of particles that we name *A*, *B* and *C*, but such that at each site there is only one particle. We prove that proper choices of density fluctuation fields (that match those from nonlinear fluctuating hydrodynamics theory) associated to the (two) conserved quantities converge, in the limit $N\rightarrow \infty$, to a system of stochastic partial differential equations, that can either be the Ornstein–Uhlenbeck equation or the Stochastic Burgers equation. To understand the cross interaction between the two conserved quantities, we derive a general version of the Riemann–Lebesgue lemma which is of independent interest.

## Introduction

One of the major open problems in statistical mechanics is the characterisation of the macroscopic evolution equations from the large-scale description of the conserved quantities in Newtonian particle systems. By replacing a deterministic dynamics with a stochastic one, many mathematical techniques have been developed in the last forty years and very interesting results have been obtained. The first class of results is related to the well-known hydrodynamic limit, which consists of deriving the space-time evolution equations for the conserved quantities of a system from the underlying random evolution of its microscopic counterpart. The second is related to the description of the fluctuations of the random microscopic system around its typical profile. In the former case, the limit is deterministic and given in terms of a solution to a PDE, the hydrodynamic equation; while in the latter, the limit is random and given in terms of a solution to a stochastic PDE. The focus of this article is on the second problem and more specifically our goal is to derive the fluctuations for a model that has *more than one* conservation law. To illustrate our results we first give a brief overview on what happens for a simplified version of our model which has only one conservation law.

### The exclusion process

One of the most studied interacting particle systems (and perhaps the most classical one) is the exclusion process [26] which was introduced in the mathematics community by Spitzer in [37]. Let us consider its evolution on the discrete one dimensional torus with *N* sites, that we denote by ${\mathbb {T}}_N$. At each site of ${\mathbb {T}}_N$ there can be at most one particle and, after an exponential clock of rate 1, particles at the bond $\{x,y\}$ exchange their positions independently at a rate given by a transition probability $p(\cdot )$. We restrict ourselves to the case in which particles only move to nearest neighbour sites so that if *z* is the size of the jump, then $p(z)=0$ if $|z|>1.$ When $p(\cdot )$ is symmetric i.e. $p(1)=p(-1)=1/2$, the system is the well known symmetric simple exclusion process (SSEP); when $p(1)=1-p(-1)=p\ne 1/2$ the system is the so-called asymmetric simple exclusion process (ASEP) and when $p(1)-p(-1)=E/N^\gamma$, $\gamma \in [0,\infty ]$, the system is the weakly asymmetric simple exclusion process (WASEP), which interpolates between the SSEP (for $\gamma =\infty$) and the ASEP (for $\gamma =0$ and $E=2p-1$). Since particles only swap positions on $\mathbb {T}_N$, the conserved quantity is the number of particles, i.e. $\sum _{x\in {\mathbb {T}}_N}\eta _t(x)$ where the variable $\eta _t(x)$ denotes the number of particles at site *x* and time *t*. From the exclusion rule $\eta _t(x)\in \{0,1\}$ and if $\eta _t(x)=1$, we say that the site *x* is occupied, and empty otherwise. The invariant measures are the Bernoulli product measures of parameter $\varrho \in (0,1)$, that indicates the density of particles.

We consider the system speeded up in the time scale $tN^a$, where $a>0$ and we define the empirical measure associated to the unique conserved quantity, i.e. the number of particles, as

$$\begin{aligned} \pi _t^{N}(du): =\frac{1}{N} \sum _{x\in {\mathbb {T}} _N} \eta _{tN^a}(x)\, \delta _{\frac{x}{N}}\left( du\right) , \end{aligned}$$

where for ${\tilde{u}}\in {\mathbb {T}}$ the notation $\delta _{\tilde{u}}(du)$ is for the Dirac measure at ${\tilde{u}}$. The hydrodynamic equation for $\pi ^{N}$ depends on the asymmetry, which in turn determines the relevant time-scale: for SSEP the time scale is diffusive, $tN^2$, and the hydrodynamic equation is the heat equation given by $\partial _t\varrho (t,u)=\tfrac{1}{2}\Delta \varrho (t,u)$; for ASEP the time scale is hyperbolic, *tN*, and the equation is the inviscid Burgers equation $\partial _t\varrho (t,u)= E\nabla F(\varrho (t,u))$, for $F(\varrho )=\varrho (1-\varrho )$; for WASEP with $\gamma =1$, the time scale is again diffusive, $tN^2$, and the equation is the viscous Burgers’ equation

$$\begin{aligned} \partial _t \varrho (t,u)=1/2\Delta \varrho (t,u)+E\nabla F(\varrho (t,u)), \end{aligned}$$

where *F* is the same as before, see [13] for a proof on the weakly asymmetric case and references therein for the other cases.

Now we turn to the description of the fluctuations around the hydrodynamic limit. The study of non-equilibrium fluctuations is usually very intricate as it requires a good knowledge of the decay of correlations of the system, which is generally difficult to have. From now on, we assume that the system starts from the Bernoulli product measure of parameter $\varrho$. We define the fluctuation field associated to the density as the linear functional defined on a smooth test function *f* as

$$\begin{aligned} {{\mathscr {Y}}^{N}_t({f})=\frac{1}{\sqrt{N}} \sum _{x\in {\mathbb {T}}_N}f(\tfrac{x}{N})(\eta _{tN^{a}}(x)-\varrho ) }. \end{aligned}$$

Once again, the strength of the asymmetry plays a crucial role in the type of limit ${\mathscr {Y}}$ we can get for ${\mathscr {Y}}^N$: for SSEP in the diffusive time scaling $tN^2$, it is given by the solution to a Ornstein–Uhlenbeck equation; for WASEP with weak asymmetry, i.e. $\gamma >1/2$, and under diffusive scaling, by the solution to a Ornstein–Uhlenbeck equation, while for $\gamma =1/2$ by the energy solution to the stochastic Burgers (SB) equation (see Definition 2.5); and finally for the ASEP in hyperbolic scaling *tN*, the fluctuations are linearly transported in time with a velocity given by $(1-2\varrho )(1-2p)$. Note that if $\varrho =1/2$, then the evolution for the limit field in ASEP in the hyperbolic time scale is trivial and the same holds even for $\varrho \ne 1/2$, as can be seen by redefining the field in a time moving frame with the velocity $(1-2\varrho )(p-q)N^{a-1}t$. To get non-trivial fluctuations we have to speed up time and choose $a=3/2$. Upon doing so the limiting field is the so-called KPZ fixed point (KPZ-fp) [33], which has been constructed in [32]. In [16] it was proved that up to the time scale $tN^{1+\delta }$ with $\delta <1/3$ there is no evolution of the field. In fact, the results of [16] applied to WASEP show that below the line $a=\tfrac{4}{3}(\gamma +1)$ the evolution is trivial, but in fact, this behaviour goes up to the line $a=\gamma +3/2$ and in all this line the limit is the KPZ-fp, see [33].

Summarising, for simple exclusion processes by changing the strength of the asymmetry, the system has either diffusive behaviour (Gaussian fluctuations, for $\gamma >1/2$), or KPZ behaviour (when $\gamma \le 1/2$) and in between, for $\gamma =1/2$, there is the SB equation or the KPZ equation introduced in [31]. In fact, these large-scale statistics can be obtained from a variety of different microscopic models and, therefore, they are universal, in the sense that the limit does not depend on the details of the underlying microscopic model.

### Universality for one component systems

To characterise the universality classes for one-component systems, let $h=h(t,u)$ be the stochastic process encoding the quantity of interest for our model (usually called height function), and *r*, *z* be two constants. We consider the space-time renormalization group operator with exponents 1 : 1/*r* : *z*/*r* given by

$$\begin{aligned} {\mathfrak {R}}_\lambda h(t,u)=\lambda ^{-1}h(t\lambda ^{z/r},u\lambda ^{-1/r}). \end{aligned}$$

A universality class consists of the basin of attraction of the limit $H:=\lim _{\lambda \rightarrow 0}{\mathfrak {R}}_\lambda h$ under the operation of rescaling ${\mathfrak {R}}_\lambda$ defined above. For one component systems, as the exclusion processes described above, several universality classes arise: the Edwards-Wilkinson (EW) class whose exponents are 1:2:4 and the super-diffusive KPZ class, whose scaling exponents are 1:2:3. The connection between these two universality classes, namely the EW and the KPZ, is the KPZ equation or the SB equation. In the case of the simple exclusion process for an asymmetry of order $O(N^{-\gamma })$, the crossover goes from diffusive behaviour, i.e. the EW class (corresponding to the phase where symmetry dominates, $\gamma >1/2$), to KPZ for $\gamma <1/2$ and time scale $a=\frac{3}{2}+\gamma$, and the transition goes through the SB (or the KPZ) equation, corresponding to the phase where both symmetry and asymmetry have the same impact $\gamma =1/2$, see [33, 40].

We highlight that the list of universality classes is not exhausted by those described above. In [10], it was introduced a temperature-dependent model which reduces to the classical ballistic deposition model at zero temperature (and thus conjectured to display KPZ-type fluctuations), but whose infinite-temperature version is a random interface whose large-scale statistics are neither EW nor KPZ. Its scaling limit is the Brownian Castle (BC), a renormalization fixed point, whose scaling exponents are 1:1:2. The BC is itself conjectured to be universal, in that any interface model which displays both horizontal and vertical fluctuations but no smoothing should belong to its basin of attraction. Moreover, while for WASEP it is the asymmetry whose tuning determines the crossover from EW to KPZ and under a suitable scaling leads to the SB equation, for the connection between BC and EW a similar role is played by the smoothing. As shown in [11], there is an uncountable family of (different) processes, the so-called $\nu$-Brownian Castles, for $\nu$ a probability measure on [0, 1], which interpolate BC and EW and therefore represent the analogue of the SB equation in this context.

### Nonlinear fluctuating hydrodynamic theory

Universality classes are identified by exponents and scaling functions that characterise the macroscopic behaviour of the fluctuations of the thermodynamical quantities of interest in a microscopic system. To see what universality classes might pop up and how the aforementioned exponents and scaling functions arise, we outline the approach of Nonlinear Fluctuating Hydrodynamics Theory (NLFH), which precisely describes the fluctuations of the conserved quantities of multi-component systems in terms of stochastic PDEs. The starting point of NLFH is the hydrodynamic scenario in a strong asymmetric regime (to make an analogy to WASEP, this corresponds to $\gamma =0$ or simply bear in mind ASEP).

Suppose that our microscopic system has a family of invariant measures parametrised by a quantity $\varrho$ and let us denote by $\mu _\varrho$ the corresponding measure, with $\langle \cdot \rangle _{\mu _\varrho }$ the average with respect to it. Assume that the hydrodynamic equation for the thermodynamical quantity of interest is (in the hyperbolic scaling *tN*) given by

$$\begin{aligned} \partial _t \varrho (t,u)+\partial _x j(t,u)=0, \end{aligned}$$

where $j(t,u)=\langle j \rangle _{\mu _{\varrho (t,u)}}$ and *j* is the instantaneous current of the system, and from now on we will always denote the derivative with respect to the first variable (i.e. time) by $\partial _t$ and $\partial _x$ that with respect to the second (i.e. space). Writing the current in terms of the density $\varrho$, then we have $\partial _x j(\varrho (t,u))= j'(\varrho (t,u))\partial _x \varrho (t,u)$ so that the previous display reads

$$\begin{aligned} \partial _t \varrho (t,u)+j'(\varrho (t,u))\partial _x\varrho (t,u)=0. \end{aligned}$$

Now we add a diffusion term $D\partial _x^2 \varrho (t,u)$ and a conservative noise $B\partial _x \xi$, where $\xi$ is a space-time white-noise. The idea now consists of expanding $\varrho (t,u)$ around its stationary value $\varrho$ as $\varrho (t,u)=\varrho +Y(t,u)$. Before proceeding, let us see what happens in ASEP. In this case the averaged current is given by $j(\varrho )=(p-q)\varrho (1-\varrho )$. For simplicity let us take $p=1$, but the same argument works for any $p\in (1/2,1]$. Then we obtain

$$\begin{aligned} \partial _t Y(t,u)=D \partial _x^2 Y(t,u)-\partial _x (1-2\varrho ) Y(t,u)+\partial _x (Y(t,u))^2-B \partial _x \xi _u(t), \end{aligned}$$

which is nothing but a stochastic Burgers equation. Note that in the equation above, the first term on the right-hand side is the diffusive term, the second is a drift that can be removed via a Galilean transformation, i.e. by looking at the system in a time dependent moving frame, and the third, the quadratic term, comes from the strong asymmetric regime. Such a term cannot be removed and is the reason why different limit behaviours are observed in the symmetric, in which this term is absent, and asymmetric cases.

For a multi-component system, we follow the same procedure and, since by the NLFH theory the universal behaviour is dictated by the quadratic term, we neglect the higher order contributions in the expansion of $\varrho (t,u)$. In other words, we expand the current-density relation up to second order and we get

$$\begin{aligned} \partial _t Y(t,u)=D\Delta Y(t,u)-j'{(\varrho )}\nabla Y(t,u)+\frac{j''(\varrho )}{2}\nabla (Y(t,u))^2-B \nabla \xi _u(t). \end{aligned}$$

As above, by changing variables $u\rightarrow u-j'(\varrho )$ (Galilean transformation) the drift term disappears and therefore we derive once more a stochastic Burgers equation.

In order to obtain a much richer diagram (than the one for the simple exclusion explained above) in which different universality classes arise and interplay non-trivially with each other, we now turn our attention to multi-component systems, which are the main focus of the present paper. More precisely, consider a system with *M* conserved quantities and let $\vec {\varrho }$ be the vector whose $\alpha$-th entry denotes the $\alpha$-th quantity, $\alpha \in \{1,2,\cdots , M\}$. The hydrodynamic equation is then given by a system of conservation laws: $\partial _t \varrho _\alpha (t,u)+\nabla j_{\alpha }(t,u)=0$, where $\alpha \in \{1,2,\cdots , M\}$. Once again, we expand the density as $\varrho _\alpha (t,u)=\varrho _\alpha +Y_\alpha (t,u)$ and express the associated current $j_\alpha (\vec {\varrho })$ as a function of $\vec {\varrho }$. Adding a diffusion and a noise term, in the form of an *M*-dimensional space-time white noise $\vec {\xi }$, and neglecting higher order contributions, we reach

$$\begin{aligned} \partial _t \vec {Y}=-\nabla \Big \{J\vec {Y}+\frac{1}{2}\sum _{\alpha =1}^M\vec {Y}^T H^\alpha \vec {Y}+D\nabla \vec {Y}+B\vec {\xi } \Big \}, \end{aligned}$$

where *J* is the jacobian of the current matrix whose entries are given by $J^\alpha _\beta =\frac{\partial j_\alpha }{\partial \varrho _\beta }$ and $\mathscr {H}^\alpha$ are the Hessians of the current matrix whose entries are given by $H^\alpha _{\beta ,\delta }=\frac{\partial ^2 j_\alpha }{\partial \varrho _\beta \partial \varrho _\delta }$. At this point we transform the fluctuation fields $\vec {Y}$ into normal fields $\vec {\phi }$ via $\vec {\phi }=R\vec {Y}$. The matrix *R* is chosen to diagonalise the Jacobian *J*, i.e. $RJR^{-1}=\textrm{diag}(v_\alpha )$, where $\{v_\alpha , {\alpha \in \{1,2,\cdots , M\}}\}$ are the eigenvalues of *J*. The evolution of $\vec {\phi }$ is then given by

1.1 $$\begin{aligned} \partial _t \phi _\alpha (t,u)=-\nabla \Big \{v_\alpha \phi _\alpha +\vec {\phi }^TG^\alpha \vec {\phi } +({\tilde{D}}\nabla \vec {\phi })_\alpha +({\tilde{B}}\vec {\xi })_\alpha \Big \} \end{aligned}$$

where ${\tilde{D}}= RDR^{-1}$, ${\tilde{B}}=RB$ and $G^\alpha$ are the coupling matrices given by

$$\begin{aligned} G^\alpha =\frac{1}{2}\sum _{\beta =1}^M R_{\alpha ,\beta }(R^{-1})^T H^\beta R^{-1}. \end{aligned}$$

Note that the SPDE in (1.1) has a drift term given by $v_\alpha$, which suggests that the normal field $\phi _\alpha$ should be taken in a moving frame with velocity $v_\alpha$.

In order to derive the universal large-scale behaviour, the NLFH looks at the structure function of $\vec {\phi }$ which is defined as

1.2 $$\begin{aligned} {S_{\alpha ,\beta }(t,u)=\langle \phi _\alpha (t,u)\phi _\beta (0,0)\rangle _{\mu _\varrho }.} \end{aligned}$$

Now, under the strict hyperbolicity condition which requires all velocities $v_\alpha$ to be different, the off-diagonal components ($\alpha \ne \beta$) of the structure function are expected to decay very fast and thus should not contribute to the asymptotic limit. The behaviour of the diagonal elements, i.e. $S_\alpha (t,u)=S_{\alpha ,\alpha }(t,u)$, should instead be given by

$$\begin{aligned} S_\alpha (t,u)\sim (C_\alpha t)^{-1/{z_\alpha }}f_\alpha \Big ((C_\alpha t)^{-1/{z_\alpha }}(u-v_\alpha t)\Big ), \end{aligned}$$

where $z_\alpha$ is a dynamical exponent and $f_\alpha$ is a scaling function. To deduce the value of $z_\alpha$ and the form of $f_\alpha$, the effect of the nonlinearity and the noise must be suitably balanced and this can be done via a memory kernel, that we now discuss. Since the normal fields solve (1.1), we see that $S_\alpha :=S_{\alpha ,\alpha }$ in (1.2) satisfies

$$\begin{aligned} \partial _t S_\alpha (t,u)=-v_\alpha \partial _x S_\alpha (t,u)+{\tilde{D}}_{\alpha ,\alpha }\partial _x^2 S_\alpha (t,u)+\int _0^t{ds} \int _{\mathbb {R}} dv S_{\alpha }(t-s,u-v) \partial _x^2M_{\alpha ,\alpha }(s,v) \end{aligned}$$

where $M_{\alpha ,\alpha }$ is the memory kernel and is given by

1.3 $$\begin{aligned} M_{\alpha ,\alpha }(s,v)=2\sum _{\beta ,\delta }(G_{\beta ,\delta }^\alpha )^2 S_\beta (s,v)S_{\delta }(s,v). \end{aligned}$$

Once again, under the strict hyperbolicity condition, the off-diagonal terms should not contribute so that the dynamical exponent $z_\alpha$ and the scaling function $f_\alpha$, and consequently the corresponding universality class, should be determined by the diagonal ones. Neglecting the off-diagonals, the memory kernel reduces to $M_{\alpha ,\alpha }(s,v)=2\sum _{\beta }(G_{\beta ,\gamma }^\alpha )^2 (S_\beta ({s,v}))^2$.

Let us now describe the possible limits. To this end, we introduce the set ${\mathbb {I}}_{\alpha }$ of those indices such that $G^\alpha _{\beta ,\beta }$ in (1.3) is not zero, i.e. ${\mathbb {I}}_{\alpha }:=\{\beta : G^\alpha _{\beta ,\beta }\ne 0\}$. For two component systems (which is the case for the model studied in this article) one can get

- Diffusive behaviour if ${\mathbb {I}}_{\alpha }=\emptyset$, i.e. the EW universality class for the field $\alpha$ corresponding to $z_\alpha =2$ and $f_\alpha$ the usual heat kernel,
- KPZ behaviour if $\alpha \in {\mathbb {I}}_{\alpha }$, corresponding to $z_\alpha =3/2$,

which are the same obtained for systems with only one conservation law (see the discussion above for WASEP). But now if for the normal field $\alpha$ the self-coupling term vanishes i.e. $G_{\alpha ,\alpha }^\alpha =0$, but for $\beta$ we have $\beta \in I_\alpha$, i.e. $G^\alpha _{\beta ,\beta }\ne 0$, then the dynamical exponent is equal to $z_\alpha =\min _{\beta \in I_\alpha }\{1+\frac{1}{z_\beta }\}$ and the scaling function is a $z_\alpha$-stable distribution given in Fourier space by

$$\begin{aligned} {\hat{S}}(t,k)=\frac{1}{\sqrt{2\pi }}\exp \Big \{-iv_\alpha tk-C_\alpha t|k|^{z_\alpha }\times (1-i A_\alpha {{\,\textrm{sgn}\,}}(k)\tan (\tfrac{\pi z_\alpha }{2}))\Big \}, \end{aligned}$$

where ${\hat{S}}$ denotes the Fourier transform given by ${\hat{S}}(t,k)=\frac{1}{\sqrt{2\pi }}\int _{\mathbb {R}} S(t,u)e^{-iku}du$, $C_\alpha$ is a constant and $A_\alpha \in [-1,1]$ an asymmetry. Now, since $z_\alpha$ satisfies $z_\alpha =\min _{\beta \in I_\alpha }\{1+\frac{1}{z_\beta }\}$, we necessarily have that for all *n*, $z_\alpha =F_{n+3}/F_{n+2}$ where $F_n$ is the Fibonacci number defined by the recursion $F_{n+2}=F_{n+1}+F_n$ and $F_1=F_2=1$. As a consequence, if in the system there is not a normal field with dynamical exponent $z_\alpha =2$ or 3/2, then the only possibility is to have $z_\alpha =\frac{1+\sqrt{5}}{2}$, the Golden number, for all the fields. The reason why only this type of $z_\alpha$-Lévy stable distributions appear with $z_\alpha$ given as above, is still mysterious. The predictions for systems with two conservation laws was first derived in [39], while for *n* conservation laws in [30].

Let us stress that the assumption that the current-density relation is strictly-hyperbolic (i.e. the velocities of the normal fields are all different) is crucial because it *formally* allows to neglect the crossed terms $S_\alpha (s,v)S_\beta (s,v)$, $\beta \ne \alpha$, and focus only on the diagonals. One of the goals of this article is to give for the first time a rigorous mathematical proof of the fact that non-diagonal terms of coupling matrices are indeed negligible. To do so, we consider a multi-component model with two conservation laws that we now describe.

### The particle exchange model

We study a generalization of the simple exclusion process by allowing three types of particles, that we name *A*, *B* and *C*. In the *ABC* model each site of the one-dimensional torus ${\mathbb {T}}_N=\mathbb {Z}/N\mathbb {Z}$ is occupied by one and only one particle, which can be of type *A*, *B* or *C*. In the *classical* *ABC* model, introduced by Evans et. al in [14, 15], particles exchange their positions with nearest neighbours particles with the asymmetric rates: $AB\rightarrow BA$, $BC\rightarrow CB$, $CA\rightarrow AC$ with rate $q=q(N)<1$ and $BA\rightarrow AB$, $CB\rightarrow BC$, $AC\rightarrow CA$ with rate 1. For this dynamics on the torus the invariant measure is explicitly known only in the case that the number of particles of each species are equal, in which case it is given by a Gibbs measure of a certain Hamiltonian having long range pair interactions. In this context, in the weakly asymmetric regime $q=1-O\left( \frac{\beta }{N}\right)$ introduced by Clincy et al. in [12], we recently obtained in [20] the system of hydrodynamic equations (with boundary conditions) that describes the evolution of the density field of each species for the dynamics in a open interval connected with reservoirs (in which case the invariant measure is not explicitly known).

**Fig. 1:**
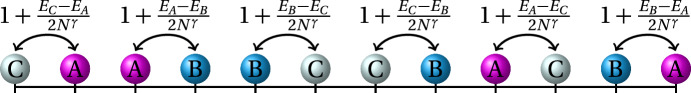
Dynamics of the model

In the present work we consider an *ABC* model with different rates. The *ABC* model is a particular case of the general *n*-component particle exchange model that is presented in [36] and, as argued therein, can also be seen as a fluctuating directed polymer in $d\ge 2$. We now assume that the interaction rates depend on three constants $E_A, E_B$ and $E_C$: for $(\alpha , \beta )\in \{A,B,C\}$ the transposition $(\alpha ,\beta )\rightarrow (\beta ,\alpha )$ occurs at rate $1+\frac{E_\alpha -E_\beta }{2N^{\gamma }}$, as it is summarised in Fig. 1 below.

It turns out, as discussed in [35], that for this model, in each irreducible class, the invariant measure is uniform over all possible configurations (see Lemma 2.1). As discussed in [8] the hydrodynamic limit for the density of particles *A* and *B*, in the diffusive time scaling $a=2$ and for $\gamma =1$, is given by the system of equations (2.9). In the present work we are interested in the fluctuations around the hydrodynamic limit.

There are three important cases to distinguish depending on the choice of rates, apart from the case $E_A=E_B=E_C$, which is trivial as the dynamics does not distinguish particles of type *A*, *B* or *C* for which the limit of the fluctuations is Ornstein–Uhlenbeck. The case **(I)** is when $E_A=E_B$ so that *A* and *B* are exchanged at rate 1. The case **(II)** is when $E_B=E_C$ and particles of type *B* and *C* are exchanged at rate 1. Let us already mention here that the fact that two types of particles exchange at rate 1 suggests that one normal field should have diffusive behaviour. And, in case **(II)** for example, if we look at the particles of type *A*, since the dynamics does not distinguish between *B* and *C*, we can conclude that the field of particles of type *A* behaves as in WASEP. The case **(III)** is the most general and all exchange rates are weakly asymmetric, with an intensity regulated by the parameter $\gamma$.

We note that the dynamics above conserves two quantities: the total number of particles of type *A* and the total number of particles of type *B*. Nevertheless any linear combination of these two quantities is again conserved. The invariant measures of this model are explicit: for any constant densities $\rho _A$, $\rho _B$ and $\rho _C=1-\rho _A-\rho _B$, the product measure $\nu _\rho$ given on $x\in {\mathbb {T}}_N$ and $\alpha \in \{A,B,C\}$ by $\nu _\rho (\eta : \eta (x)=\alpha )=\rho _\alpha$, is an invariant measure. Since the dynamics of each type of particle depends on the other, the evolution equations are not closed and it is a priori unclear how to derive the limiting equations. Nevertheless, according to NLFH, the normal fields can be identified and predictions can be made (see Appendix A, in which this is done in detail).

Alternatively, to identify the normal fields we can (and will) proceed as follows. First, we analyse the action of the infinitesimal generator on the occupation variables for particles of types *A* and *B*, derive their instantaneous currents and centre all variables. This then will allow us to evaluate the generator on a generic field given by a linear combination of the centred occupation variables for *A* and *B*. The expression we obtain will display drift terms that blow up in the limit and we force these to be zero by passing the generic field to a time moving frame. At this point, the derivation of the correct normal fields boils downs to solving a system of two equations with two unknowns: the velocity of the moving frame and the constant defining the linear combination of fields (in principle the constants are two, but, by linearity, one can always fix one of them to be equal to one). This procedure is carried out in Sect. 3 and delivers the same normal fields as those predicted by the NLFH theory.

Once blowing up terms are removed from the evolution equations, we are left with higher order terms which, in this specific model, are quadratic and can be written as products of the occupation variables for particles of type *A* or *B*. The coefficients in front of these quadratic terms are nothing but the entries the coupling matrices.

**Fig. 2:**
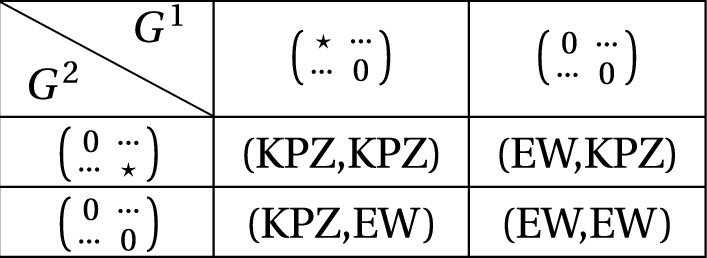
Classification of the universal behaviour of the two modes observed in the ABC model by the structure of the mode coupling matrices. A star denotes a non-zero entry, a dot represents an arbitrary value

For the multi-species WASEP we consider, the special structure of these coupling matrices implies that for the normal fields, we always have $G^\alpha _{\beta ,\beta }=G^\beta _{\alpha ,\alpha }=0$ whatever the choice of the constants $E_\alpha$ is (see the computations in Appendix A). This means that the only important contribution comes from self-coupling terms (i.e. the entries $G^\alpha _{\alpha ,\alpha }$ and $G^\beta _{\beta ,\beta }$). Therefore the predicted limit behaviour from NLFH (in the strong asymmetric regime, i.e. $\gamma =0$) is either diffusive (the EW universality class) or KPZ behaviour (in the KPZ universality class), depending on whether $G^\alpha _{\alpha ,\alpha }$ or $G^\beta _{\beta ,\beta }$ is zero or not. See Fig. 2 for a summary of the predictions.

### Our contribution

The main contribution of this article is twofold. First, we rigorously determine the large-scale behaviour of the non-diagonal terms of the coupling matrices. In our context, this means that, the quadratic terms due to crossed products of normal modes associated to particles of type *A* and *B*, *only interact via their initial total mass* and *do not produce any additional non-linear term in the limit*. The result is shown in wide generality as its assumptions only impose mild conditions on the moments of the fields, so that in particular it holds irrespectively of the specific dynamics one considers and even allows for products of more than two fields, but we are restricted to the diffusive time scale. Let us stress that, while on the one hand this puts on firm ground the assumption in NLFH that off-diagonal terms are negligible (at least in diffusive time scale), on the other we *do observe* a contribution which is though trivial as it only involves their initial total masses (which are conserved quantities of the system). This is something which was not predicted by mode coupling because it only looks at the fluctuations of the normal modes at a fixed time.

Second, we show that, in the two component model we consider, if the asymmetry is weak, $\gamma >1/2$, then the limit of both fields is again diffusive, so the system falls in the EW universality class. More interestingly, we carry out a full analysis of the case $\gamma =1/2$, in which both the EW and the KPZ behaviour arises. To be precise, we prove that any normal field whose coupling matrices has only non-zero non-diagonal entries, has again diffusive behaviour. From this result we then show that in cases **(I)** and **(II)** one mode is diffusive while the other is KPZ. For the case **(III)**, we show instead that both fields have indeed KPZ behaviour (since we are restricted to the choice $\gamma =1/2$, and when we say KPZ behaviour, we mean that the fluctuations are given in terms of the SB equation). In other words, we rigorously prove that, for $\gamma =1/2$, *all the predicted results summarised in Fig.* 2*hold true*.

Let us stress that since the development of NLFH theory very little advances have been made for multi-component systems (for results on the Lévy limits with parameters 3/2 or 5/3 see [2–6, 9, 17, 25, 34]). For some models (as, for example, chain of oscillators with the Fermi–Pasta–Ulam potential), even the understanding of the correct linear combinations of fields has not been successfully addressed (apart from numerical simulations). Up to our knowledge, prior to this work, only the case of diffusive behaviour, Lévy-3/2 and Lévy-5/3 have been derived rigorously. This is the first time in which diffusion/KPZ, as well as KPZ for both modes has been obtained. In [1] systems of coupled KPZ equations appear but the strict hyperbolicity condition was lacking, so that, contrary to our case, the resulting equations are coupled.

Let us now comment on the technical aspects of our work. To obtain the KPZ behaviour we extend the second order Boltzmann–Gibbs principle derived in [18, 19] to the setting of multi-component systems. The Boltzmann–Gibbs principle allows to replace products of occupation variables with uniform averages in big microscopic boxes, which then enables us to close the equations for each of the normal fields and ultimately identify the limit as energy solutions of the SB equation. In order to prove that the contribution from crossed terms is negligible, we need a further refinement of the aforementioned principle, according to which, instead of taking uniform averages, we suitably distribute the mass in such a way that, in the macro-limit, this can be well approximated by a smooth function. Once this is in place, the control over the crossed term boils down to rewrite it in Fourier and apply a version of Riemann–Lebesgue lemma for stochastic processes which satisfy suitable moment bounds. Here, the strict hyperbolicity condition is essential, as if it fails the crossed terms might survive in the limit (see e.g. the zero-range model studied in [1] where this indeed happens).

To conclude this introduction, we mention future work and possible extensions of our result. The first important open problem is to go beyond the diffusive time scale and to show that in fact the results presented in the table above can be obtained in the whole range of the parameter $\gamma >0$. Moreover, it would also be interesting to push forward our results when the system is evolving in the infinite lattice or in the open interval but in the presence of a Glauber dynamics at the boundary.

Another possible direction is to consider particle exchange models in which more than one particle per site is allowed. We believe that in this case the scenario could be richer and possibly other universal behaviours might appear. At last, it would be very interesting to apply our results to Hamiltonian models as the chain of oscillators and that in [7]. Our contributions might provide useful insights which could help to derive the normal fields and determine their large-scale behaviour.

### Outline

In Sect. 2 we introduce the model and we state our main result. In Sect. 3 we present the computations for the martingales at the discrete level for all the cases defining the jump rates. In Sect. 4 we give the proof of the limit of the fluctuation fields. To do so in Subsection 4.1 we prove that the sequence of fluctuation fields is tight and in Subsection 4.2 we characterize the limit fields either as solutions to Ornstein-Uhlenbeck equations or as energy solutions to the stochastic Burgers equation. In Sect. 5 we prove that the contribution of crossed terms are negligible in the asymptotic limit and Sect. 6 is devoted to the proof of second order Boltzmann Gibbs principles. In the Appendix A we present the computations of NLFH theory for our specific model and in Appendix B we present some auxiliary results.

## Statement of results

### The model

We consider the discrete ring with *N* sites, ${\mathbb {T}}_N=\mathbb {Z}/N\mathbb {Z}$; each site is occupied by exactly one particle, and such particle is of species $\alpha$ with $\alpha \in \{A,B,C\}$. The system evolves by nearest-neighbour exchanges of particles in the presence of an external field that interacts with particles of different species with different strength denoted by $E_A,E_B,E_C$.

The space of configurations is ${\Omega }_{N}=\{A,\,B,\,C\}^{{\mathbb {T}}_N}$; its elements are denoted with $\eta$; on each site $x\in {\mathbb {T}}_N$, $\eta (x)\in \{A,\,B,\,C\}$. We define the occupation numbers of the species $\alpha \in \{A,\,B,\,C\}$ as the function $\xi ^{\alpha }:{\Omega }_{N}\rightarrow \{0,\,1\}^{{\mathbb {T}}_N}$ acting on the configurations in the following wayNote that $\sum _{\alpha }\xi ^{\alpha }_x(\eta )=1$ for all $x\in {\mathbb {T}}_N$ and for all $\eta \in \Omega _N$. We consider a *weakly asymmetric regime*: for a configuration $\eta \in \Omega _N$ and $x\in {\mathbb {T}}_N$ such that $(\eta _x,\eta _{x+1})=(\alpha ,\beta )$ for $\alpha ,\beta \in \{A,\,B,\,C\}$, $\alpha \ne \beta$, the exchange to $(\eta _x,\eta _{x+1})=(\beta ,\alpha )$ occurs at rate

$$\begin{aligned} c^{\alpha ,\beta }:=c^{\alpha ,\beta }_{N,\gamma }=1+\frac{E_\alpha -E_\beta }{2N^\gamma }. \end{aligned}$$

The total number of particles of each species, $N_\alpha (\eta )=\sum _{x\in {\mathbb {T}}_N}\xi ^\alpha _x(\eta )$, $\alpha \in \{A,\,B,\,C\}$, is conserved and $N_{A}+N_{B}+N_{C}=N$.

Given two sites $x,\,y\in \mathbb {T}_{N}$ and given a configuration $\eta \in {\Omega }_{N}$, we define $\eta ^{x,y}$ as the configuration obtained by exchanging particles on sites *x* and *y*, i.e.

$$\begin{aligned} \eta ^{x,y}(z)={\left\{ \begin{array}{ll} \eta (y) &  z=x\\ \eta (x) &  z=y\\ \eta (z) &  \text {otherwise}. \end{array}\right. } \end{aligned}$$

Thus we can define the process as a continuous time Markov chain with state space ${\Omega }_{N}$ and generator $L_{N}$ acting on the functions $f:{\Omega }_{N}\rightarrow {\mathbb {R}}$ as

2.1 $$\begin{aligned} L_{N}f(\eta )=N^a\sum _{x\in {\mathbb {T}}_N}c_x(\eta )[f(\eta ^{x,x+1})-f(\eta )], \end{aligned}$$

with

2.2 $$\begin{aligned} c_x(\eta )=\sum _{\alpha ,\beta }c^{\alpha ,\beta }\xi ^\alpha _x\xi ^\beta _{x+1} \end{aligned}$$

where the sum runs over $\alpha \ne \beta \in \{A,B,C\}$. The dynamics is summarized in Fig. 1.

In (2.1), $a>0$, but everywhere in the article we will assume that $a=2$, i.e. the process will be speeded up in the diffusive time scale and this choice will be justified ahead (see e.g. Lemma 3.1).

Note that the rates satisfy the pairwise balance^1^

2.3 $$\begin{aligned} c^{AB}+c^{BC}+c^{CA}=c^{BA}+c^{CB}+c^{AC}. \end{aligned}$$

In [35] a criterion to identify the invariant measure for generalised exclusion processes satisfying the above was showed.

#### Lemma 2.1

Under condition (2.3), any measure $\mu _N$ on $\Omega _N$ such that

2.4 $$\begin{aligned} \mu _N(\eta ^{x,x+1})=\mu _N(\eta ), \,\text { for all }\,\eta \in \Omega _N \,\text { and all }\,x\in {\mathbb {T}}_N, \end{aligned}$$

is invariant for the dynamics with generator $L_N$.

#### Proof

For two configurations $\eta ,{\tilde{\eta }} \in \Omega _N$ denote by $R( \eta , {\tilde{\eta }})$ the jump rate from $\eta$ to ${\tilde{\eta }}$ and by $\lambda (\eta )$ the total jump rate from $\eta$, so that $\lambda (\eta )=\sum _{{\tilde{\eta }}}R(\eta ,{\tilde{\eta }})$. The measure $\mu _N$ is invariant if, for all $\eta \in \Omega _N$,

2.5 $$\begin{aligned} \sum _{{\tilde{\eta }}\ne \eta }\mu _N({\tilde{\eta }})R({\tilde{\eta }}, \eta )-\mu _N(\eta )\lambda (\eta )=0. \end{aligned}$$

Under (2.4), the last condition is equivalent to

2.6 $$\begin{aligned} \sum _{{\tilde{\eta }}\ne \eta }R({\tilde{\eta }},\eta )=\sum _{{\tilde{\eta }}\ne \eta }R(\eta ,{\tilde{\eta }}). \end{aligned}$$

For $\alpha ,\beta \in \{A,B,C\}$ and $\eta \in \Omega _N$, let  and note that the number of particles of type $\alpha$ is equal to $\sum _{\beta }N_{\alpha ,\beta }=\sum _{\beta }N_{\beta ,\alpha }$. This relation can be rewritten as

2.7 $$\begin{aligned} N_{AB}-N_{BA}=N_{BC}-N_{CB}=N_{CA}-N_{AC}. \end{aligned}$$

Noting that

$$\begin{aligned}\begin{aligned} \sum _{{\tilde{\eta }}\ne \eta }R(\tilde{\eta },\eta )&=N_{AB}(\eta )c^{BA}+N_{BA}(\eta )c^{AB}+N_{BC}(\eta )c^{CB}+N_{CB}(\eta )c^{CB}\\  &\quad +N_{CA}(\eta )c^{AC}+N_{AC}(\eta )c^{CA},\\ \sum _{{\tilde{\eta }}\ne \eta }R( \eta ,{\tilde{\eta }})&=N_{AB}(\eta )c^{AB}+N_{BA}(\eta )c^{BA}+N_{BC}(\eta )c^{BC}+N_{CB}(\eta )c^{CB}\\  &\quad +N_{CA}(\eta )c^{CA}+N_{AC}(\eta )c^{AC}, \end{aligned} \end{aligned}$$

we rewrite (2.6) as

$$\begin{aligned}&(N_{BA}-N_{AB})[c^{AB}-c^{BA}]+(N_{CB}-N_{BC})[c^{BC}-c^{CB}]\\  &\quad +(N_{AC}-N_{CA})[c^{CA}-c^{AC}]=0, \end{aligned}$$

which is true thanks to (2.7) and (2.3). $\square$

From this it follows that, when the number of particles of each species is fixed, $n_A+n_B+n_C=N$, the canonical distribution is uniform on $\{\eta \in \Omega _N: N_A(\eta )=n_A,N_B(\eta )=n_B,N_C(\eta )=n_c\}$ with

2.8 $$\begin{aligned} \mu _N(\eta )=\begin{pmatrix} N\\ n_A,\, n_B,\, n_C \end{pmatrix}^{-1}=\frac{n_A!\, n_B!\, n_C!}{N!}. \end{aligned}$$

Moreover, for any constant densities $\rho _A$, $\rho _B$ and $\rho _C=1-\rho _A-\rho _B$, the product measure $\nu _\rho$ with $\rho =(\rho _A, \rho _B, \rho _C)$, over $x\in {\mathbb {T}}_N$, such that for all $x\in {\mathbb {T}}_N$ and $\alpha \in \{A,B,C\}$, $\nu _\rho (\eta : \eta (x)=\alpha )=\rho _\alpha$, is an invariant measure on $\Omega _N$.

In the diffusive time scaling $a=2$ and for $\gamma =1$, the hydrodynamic equations for the densities of particles *A* and *B* are given by^2^

2.9 $$\begin{aligned} \partial _t \begin{pmatrix}\rho ^A\\ \rho ^B \end{pmatrix}=\Delta \begin{pmatrix}\rho ^A\\ \rho ^B \end{pmatrix}-\nabla \left( \chi (\rho )\cdot g_E \right) , \end{aligned}$$

where

$$\begin{aligned} \chi (\rho )=\begin{pmatrix} \rho ^A(1-\rho ^A) &  -\rho ^A\rho ^B\\ -\rho ^A\rho ^B &  \rho ^B(1-\rho ^B) \end{pmatrix} \end{aligned}$$

is the mobility and $$g_E=\begin{pmatrix}E_A-E_C\\ E_B-E_C\end{pmatrix}$$. The density of particles *C* can be recovered by $\rho ^C=1-\rho ^A-\rho ^B$.

### Fluctuation fields

Let us denote by ${\mathbb {T}}=[0,1)$ the continuous torus and by  the space of $\mathbb {R}$-valued smooth functions on ${\mathbb {T}}$. We define the density fluctuation fields  as2.10where $\alpha \in \{A,B,C\}$ and  is the expectation with respect to the invariant product measure associated to constant profiles. To simplify the presentation, we assume $\rho _A=\rho _B=\rho _C=\rho =1/3$ throughout the article (so that trivially for any $\alpha \in \{A,B,C\}$, ). The results for general densities can be derived from ours by taking suitable Galilean transformations, which centre the fields with respect to appropriate reference moving frames.

To lighten the notation, for $s\in [0,T]$, we suppress the dependence on $\eta _s$ from $\xi ^\alpha$ and write $\xi ^\alpha _x(s):=\xi ^\alpha _x(\eta _s)$. For $v\in {\mathbb {R}}$ and $t\ge 0$, we denote by $T_{v t}$ the translation operator acting on  as

2.11 $$\begin{aligned} T_{v t}f\left( \frac{x}{N}\right) =f\left( \frac{x-v t}{N}\right) . \end{aligned}$$

The large scale fluctuations of the density fields depend on the relation among the coupling constants $E_A, E_B$ and $E_C$, and in particular on whether they are all different or not. This leads us to distinguish some cases that we now spell out.

**Case (I):** $$E_{A}=E_B$$

In this first case, the fields that should be considered are2.12with $v_\pm =\pm \frac{(E_A-E_C)}{3N^\gamma }$ and $b=a$.

**Case (II):** $$E_{B}=E_C$$

Now instead we should look at the fields2.13with $v_\pm =\pm \frac{(E_C-E_A)}{3N^\gamma }$ and $b=a$.

#### Remark 2.2

The fields for the case $E_A=E_C$ are the same as for the Case **(II)** with *A* and *B* interchanged.

**Case (III):** $$E_{B}-E_{A}\ne E_{C}-E_{A}\ne 0$$

In this case, we have2.14where again $b=a$,

2.15 $$\begin{aligned} v_\pm =\pm \frac{\delta }{2N^\gamma }\qquad \text {and}\qquad c_\pm {:}{=}E_A-E_B\pm \tfrac{3}{2}\delta . \end{aligned}$$

Here $\delta$ is given by

2.16 $$\begin{aligned} \delta {:}{=} \tfrac{2}{3}\sqrt{(E_A-E_C)^2+(E_B-E_C)^2-(E_A-E_C)(E_B-E_C)}. \end{aligned}$$

#### Remark 2.3

Note that the completely symmetric case $E_A=E_B=E_C$ is contained in Cases **(I)** and **(II)**, and observe that in this case $v_{\pm }=0$.

#### Remark 2.4

Case **(II)** can be obtained as a consequence of Case **(I)**, looking at the pair  instead of  and recalling the relation . For this reason, we will only explicitly treat cases **(I)** and **(III)**.

Before stating the result concerning the fluctuations of the density fields, let us introduce the notion of solution for the stochastic Burgers equation and the Ornstein–Uhlenbeck equation, as they are the equations describing the limiting behaviour. For the former, we follow the definition of *energy solutions* proposed in [19].

### Energy solution to the stochastic Burgers equation

Let us recall that the stochastic Burgers equation is the SPDE given by2.17where $\lambda \in {\mathbb {R}}$, $\sigma \ne 0$ and $m\in \mathbb {R}$ are constant (and we allow *m* to be random), and  is a -valued Brownian motion with covariance given on  by2.18Above  denotes the space of ${\mathbb {R}}$-valued distributions on ${\mathbb {T}}$. The notion of solution to (2.17) we will use, is that of *energy solutions*, first proposed in [18], which allows to make sense of the non-linearity in its expression. To see how this works, let $\left\{ \overleftarrow{\iota _{\varepsilon }}\,, \overrightarrow{\iota _{\varepsilon }}; \varepsilon \in (0,1)\right\}$ be approximations of the identity respectively over intervals to the left or to right of 0, and . We define the process  as2.19where $*$ denotes the convolution operator. In the context of energy solutions, the non-linear term in the stochastic Burgers equation is interpreted as the limit for $\varepsilon \rightarrow 0$ of the process . If the process  satisfies an *energy estimate*, i.e. if there exists a finite constant $C>0$ such that for any $0\le s \le t \le T$, any $0<\delta \le \varepsilon <1$ and any , we have2.20where $\Vert \cdot \Vert _2$ is the usual $L^2(\mathbb {T})$-norm, then the limit2.21exists in $L^2$ and does not depend on the choice of $\left\{ \overleftarrow{\iota _{\varepsilon }}\,, \overrightarrow{\iota _{\varepsilon }}; \varepsilon \in (0,1)\right\}$ (so that in particular, we could have chosen $\overleftarrow{\iota _{\varepsilon }}=\overrightarrow{\iota _{\varepsilon }}$). Hence, we can postulate that the integral in time of the square of the distribution-valued process  is given by  and pose the Cauchy problem for the stochastic Burgers equation. Nevertheless, the energy estimate (2.20) alone does not guarantee uniqueness so it will not be sufficient to prove convergence of our sequence of density fields. To overcome the issue, in [21], the authors imposed an extra condition on the reversed problem, which then led to the proof of uniqueness in [22]. This extra condition consists of requiring the reversed process to satisfy the same martingale problem as the original process, but with respect to the adjoint generator. Let us remark that this is very natural (and easy to check) in the context of interacting particle systems, since it simply corresponds to considering the same process but with reversed rates.

We are now ready to characterise the solution to the stochastic Burgers equation with the following definition. Let  (resp.  be the space of continuous (resp. càdlàg) functions defined on [0, *T*] and taking values in .

#### Definition 2.5

 We say that a process  with trajectories in  is a *stationary energy solution* of the stochastic Burgers equation given in (2.17) if

(i) For each $t \in [0, T]$ the -valued random variable  is a white noise of covariance $\sigma ^{2}$;
(ii) The process  satisfies the energy estimate (2.20);
(iii) For any  and $t\in [0,T]$, the process 2.22 is a continuous martingale with respect to the natural filtration associated to , of quadratic variation  Above the process  is obtained as the $L^2$-limit (2.21);
(iv) The reversed processes  also satisfy (iii) with $\lambda$ replaced by $-\lambda$.

#### Definition 2.6

We say that  is a stationary solution of the Ornstein–Uhlenbeck equationif it satisfies conditions (i) and (iii) in Definition 2.5 with $\lambda =0$.

In the next theorem, we state existence and uniqueness for the energy solutions to (2.17), whose existence was derived in [18, 21] and the uniqueness in [22].

#### Theorem 2.7

[22, Theorem 2.4] For any value of $\lambda \in \mathbb {R}$, $\sigma \ne 0$ and (random) constant $m\in \mathbb {R}$, the stochastic Burgers equation (2.17) has a stationary energy solution, given in Definition 2.5, which is unique up to indistinguishability.

#### Remark 2.8

With respect to [18, 21, 22], the SBE we are considering features an additional transport term which is linear and therefore does not alter the existence/uniqueness theory established therein.

### Main result

Now we are ready to state the main results of this article, which are summarised in the next theorem.

#### Theorem 2.9

In the diffusive time scaling $a=b=2$, the sequence of processes , whose definition depends on the values of the constants $E_A,E_B$ and $E_C$ and is given in Sect. 2.2, converges in law in  to , where  and  are stationary solutions of stochastic Burgers equations of the form2.23started with independent initial conditions, where  and  are independent -valued Brownian motions with covariances given by (2.18) and the coefficients $m_{\pm }$, $\lambda _\pm$, $\sigma ^2_\pm$ are given by

**Case (I-II)**: $\sigma ^2_+=\tfrac{2}{3}$ and $\sigma ^2_-=\tfrac{2}{9}$.

(i) When $\gamma >1/2$, $\lambda _+=\lambda _-=0{=m_-=m_+}$, so that  are the unique solutions to the corresponding Ornstein–Uhlenbeck equations,
(ii) When $\gamma =\tfrac{1}{2}$, $\lambda _+=0$ and  where  is random and given by the total mass of the initial condition of  , while $\lambda _-=E_C-E_A$ and $m_-=0$.

**Case (III)**: $\sigma ^2_\pm =\tfrac{2}{9} (1+\frac{c_\mp ^2}{(E_A-E_C)^2}-\frac{c_\mp }{(E_A-E_C)})$, for $c_\pm$ defined according to (2.15), and

(i) When $$\gamma >1/2$$, $$\lambda _+=\lambda _-=0{=m_-=m_+}$$,
(ii) When $\gamma =1/2$, $\lambda _+=-\tfrac{E_A-E_C}{3\delta }(c_+-E_B+E_C)$ and , while $\lambda _-=-\tfrac{E_A-E_C}{3\delta }(E_B-E_C-c_-)$ and , where, as above,  is the total mass of the initial condition.

#### Remark 2.10

The additional massive transport term in (2.23) should not come as a surprise as the total mass of the initial condition is a conserved quantity of the system (thus independent of time) and *does not see* the diverging velocity. To witness, let $t\mapsto \eta _t$ be the standard periodic SSEP in diffusive time scale and evolving on $\mathbb {T}_N$ started from the Bernoulli product measure with parameter $\rho \in (0,1)$ and  be its density fluctuation field. Let *f* be a smooth non-negative non-zero periodic function and ${\bar{f}}=\int _\mathbb {T}f(u)\text {d}u$. Notice that for any $t>0$ and $\alpha >1$ we havewhere  denotes the field  tested against the function constantly equal to 1. Now, by Theorem 5.1 below, the first term converges to 0. For the other,  is nothing but the total mass of the fluctuation field which is a conserved quantity of the system and therefore independent of time. In particular, for every *s*,  and the latter converges in law to a Gaussian random variable  with variance $\rho (1-\rho )$. Therefore, we have showed thatin probability. In other words, even though we look at the process in the wrong time-frame the integral in time of the field does not fully vanish but leaves as a trace its total mass.

#### Remark 2.11

Note that the transport term in (2.23) only appears for $\gamma =1/2$ and we could have avoided its presence by an additional Galilean transformation, i.e. by modifying the definition of the velocities in Sect. 2.2. More precisely, one can show that upon setting

- In **Case (I-II)**  and ${\tilde{v}}_-:=v_-$,
- In **Case (III)** ,

the sequence  defined as in Sect. 2.2 but with ${\tilde{v}}_\pm$ instead of $v_\pm$, converges to the couple  solving (2.23) with $m_\pm = 0$ and the same choice of the parameters $\lambda _\pm$ and $\sigma _\pm$ as in the above statement. Further, the limiting processes  can be proven to satisfy the cylinder martingale problem of [23, Section 5.1], and are therefore independent.

That said, the velocities ${\tilde{v}}_\pm$ are random and their definition looks rather artificial, at least at first sight. Moreover, we believe that the observation that these transport terms appear is interesting in its own right so that we preferred to present Theorem 2.9 as stated.

#### Remark 2.12

Note, in particular, from Case **(I)** or **(II)**, that in the completely symmetric cases $E_A=E_B=E_C$, we have that $\lambda _{+}=\lambda _-=0{=m_-=m_+}$ and so, as expected,  are solutions of the Ornstein–Uhlenbeck equation, as in the SSEP.

The strategy of proof of the previous theorem consists of first showing tightness of the sequence of fields to be analysed, with respect to the Skorokhod topoplogy on  (see Sect. 4.1). From this together with Prokhorov’s theorem, the processes converge along subsequences. In order to characterise the limit uniquely, by Theorem 2.7, it suffices to show that any limit point either satisfies Definition 2.6 or points (i)-(iv) of Definition 2.5. In this latter case, we follow the strategy of [18], which has since been applied to many different models, but here we generalise it to the setting in which the system has several conservation laws. When instead the limit is a solution to the Ornstein-Uhlenbeck equation, there are two cases to distinguish. If $\gamma >1/2$, the asymmetry becomes negligible and the limiting fields evolve independently, therefore the proof follows by adapting standard arguments. If instead $\gamma =1/2$, the evolution of each field depends non-linearly on the other and therefore novel tools are needed in order to show that any crossed term ultimately vanishes (see Sect. 5).

In the next section, via Dynkin’s formula, we derive a collection of martingales for each of the cases **(I)-(III)**. These martingales provide a weak formulation for the dynamics of our process and represent the building blocks of our arguments.

## Associated martingales

In order to prove Theorem 2.9, we study the microscopic dynamics of the fields in each of the cases ** (I)**, **(II)** and **(III)**. To do so in the greatest possible generality, we start by presenting some algebraic computations which are the basis for the derivation of the martingale terms in point (iii) of Definition 2.5. Recall (2.10). We consider now a generic field  given by a linear combination of  and , i.e.3.1where $D_1, D_2,b>0$ and $v\in {\mathbb {R}}$ will be fixed later. From Dynkin’s formula, see e.g. [27, Appendix A.1.5], we know that for 3.2is a martingale with respect to the natural filtration of the process. It is (tedious but) not hard to see that the quadratic variation of  is given by3.3In the next lemma, we show that the expectation of the quadratic variation converges to a non-zero finite constant *only if* the scaling chosen is diffusive, which means that *a* must necessarily be equal to 2.

### Lemma 3.1

For , let  be the martingale in (3.2). Then, under the diffusive scaling $a=2$3.4

### Proof

Note first that for any $x\in {\mathbb {T}}_N$,

3.5 $$\begin{aligned} {\mathbb {E}}_{\nu _\rho }\big [c_x(\eta )[(D_1\xi ^{A}_{x+1}+D_2\xi ^{B}_{x+1})-(D_1\xi ^{A}_{x}+D_2\xi ^{B}_{x})]^2\big ]=\frac{4}{9}(D_1^2+D_2^2-D_1D_2).\nonumber \\ \end{aligned}$$

To prove (3.5), recall the definition of $c_x(\eta )$ in (2.2), and note that for $\alpha , \beta \in \{A,B,C\}$ such that $\alpha \ne \beta$, it holds $c^{\alpha ,\beta }+c^{\beta ,\alpha }=2.$ The left-hand side of (3.5) can be written as3.6By the exclusion rule $\xi ^A_{y}\xi ^B_y=0$, so that the second line of (3.6) is equal to zero. The last line of (3.6) is also equal to zero because $c^{A,A}=c^{B,B}=0$, since there are no exchanges between particles of the same type. The third line of (3.6) is equal towhile the first is given byfrom which (3.5) follows.

Using (3.3) and (3.5) a simple computation proves the statement. $\square$

We turn our attention to the right-hand side of (3.2) and we compute explicitly the expression inside the time integral. Observe that3.7Moreover, to determine  we need to understand the action of the generator on each type of particle, i.e. $L_N \xi ^A_x$ and $L_N \xi ^{B}_x$. It is immediate to see that $L_N \xi ^A_x=N^a(j_{x-1,x}^A-j_{x,x+1}^{A})$, where, for particles of species *A*, the infinitesimal current is given by

3.8 $$\begin{aligned} \begin{aligned} j^A_{x,x+1}&=\xi ^{A}_{x}-\xi ^{A}_{x+1}+\frac{E_{A}-E_{B}}{2N^\gamma }(\xi ^{A}_{x}\xi ^{B}_{x+1}+\xi ^{B}_{x}\xi ^{A}_{x+1})\\&\quad +\frac{E_{A}-E_{C}}{2N^\gamma }(\xi ^{A}_{x+1}+\xi ^{A}_{x}-2\xi ^{A}_{x}\xi ^{A}_{x+1}-\xi ^{A}_{x}\xi ^{B}_{x+1}-\xi ^{B}_{x}\xi ^{A}_{x+1}), \end{aligned} \end{aligned}$$

while $L_N \xi ^{B}_x=j_{x-1,x}^{B}-j_{x,x+1}^{B}$ and the infinitesimal current for particles of species *B* is given by the same expression with *A* and *B* interchanged.

We now look at the infinitesimal current $\bar{j}^\alpha$, $\alpha \in \{A,B\}$ for the centred variables , $x\in \mathbb {T}_N$, which is

3.9 $$\begin{aligned} \begin{aligned} \bar{j}^A_{x,x+1}&={\bar{\xi }}^A_{x}-{\bar{\xi }}^A_{x+1}-\frac{E_{B}-E_{A}}{6N^\gamma }({\bar{\xi }}^{A}_{x}+{\bar{\xi }}^{A}_{x+1})-\frac{E_{B}-E_{C}}{6N^\gamma }({\bar{\xi }}^{B}_{x}+{\bar{\xi }}^{B}_{x+1}) \\&\quad +\frac{E_{C}-E_{A}}{N^\gamma }{\bar{\xi }}^A_x{\bar{\xi }}^A_{x+1}-\frac{E_{B}-E_{C}}{2N^\gamma }( {\bar{\xi }}^{A}_{x}{\bar{\xi }}^{B}_{x+1}+{\bar{\xi }}^{B}_{x}{\bar{\xi }}^{A}_{x+1}) \end{aligned} \end{aligned}$$

and

3.10 $$\begin{aligned} \begin{aligned} \bar{j}^{B}_{x,x+1}&={\bar{\xi }}^{B}_{x}-{\bar{\xi }}^{B}_{x+1}+\frac{E_{B}-E_{A}}{6N^\gamma }({\bar{\xi }}^{B}_{x}+{\bar{\xi }}^{B}_{x+1})-\frac{E_{A}-E_{C}}{6N^\gamma }({\bar{\xi }}^{A}_{x}+{\bar{\xi }}^{A}_{x+1})\\&\quad +\frac{E_{C}-E_{B}}{N^\gamma }{\bar{\xi }}^{B}_x{\bar{\xi }}^{B}_{x+1}-\frac{E_{A}-E_{C}}{2N^\gamma }( {\bar{\xi }}^{A}_{x}{\bar{\xi }}^{B}_{x+1}+{\bar{\xi }}^{B}_{x}{\bar{\xi }}^{A}_{x+1}). \end{aligned} \end{aligned}$$

Thanks to the previous, a simple, but long computation, shows that for ,3.11Above, the discrete Laplacian and the discrete derivative operators are defined, for $x\in {\mathbb {T}}_N$, by

3.12 $$\begin{aligned} \begin{aligned} \Delta _N f\left( \frac{x}{N}\right)&=N^2\left\{ f\left( \frac{x+1}{N}\right) -2f\left( \frac{x}{N}\right) +f\left( \frac{x-1}{N}\right) \right\} \\ \nabla _N f\left( \frac{x}{N}\right)&=N\left\{ f\left( \frac{x+1}{N}\right) -f\left( \frac{x}{N}\right) \right\} . \end{aligned} \end{aligned}$$

### Finding the fluctuation fields

In view of Lemma 3.1, let us fix the diffusive scaling $a=2$. First we observe that, independently of the values of $D_1,D_2,v$ and *b*, the variance of the terms in the sixth and seventh lines in (3.11) are of order $O(N^{-2\gamma })$, so that they can be neglected for any $\gamma >0$. On the other hand, one sees that the variance of the terms in the fourth and fifth lines of (3.11) are of order $O(N^{2-2\gamma })$, which explodes for any $\gamma \in (0,1)$, while that on the eighth is of order $O(N^{2b-3})$. Therefore, we are forced to choose the velocity *v*, the scaling *b* and the constants $D_1$ and $D_2$, in order to annihilate them. Note that, after a replacement of discrete derivatives by continuous ones, which can be done by paying a price of a lower order with respect to *N* their sum is

3.13 $$\begin{aligned} \begin{aligned}&\Big \{N^{1/2-\gamma }\Big (D_1\frac{E_{B}-E_A}{3}+ D_2\frac{E_A-E_{C}}{3}\Big )+N^{b-3/2} D_1 v\Big \}\\&\quad \times \int _0^t \text {d}s \sum _{x\in {\mathbb {T}}_N}\nabla T_{v N^bs}f\left( \frac{x}{N}\right) {{\bar{\xi }}}^{A}_{x}(s)\\&+\Big \{N^{1/2-\gamma }\Big (D_1\frac{E_{B}-E_{C}}{3}- D_2\frac{E_{B}-E_A}{3}\Big )+N^{b-3/2} D_2 v\Big \}\\&\quad \int _0^t \text {d}s \sum _{x\in {\mathbb {T}}_N}\nabla T_{v N^bs}f\left( \frac{x}{N}\right) {{\bar{\xi }}}^{B}_{x}(s).\end{aligned} \end{aligned}$$

For the coefficients of the integrals to be equal to zero, we first want to pick *b* in such a way that the summands are of the same order, which imposes $b=a=2$, and then need to find constants $D_1, D_2$ and *v* satisfying the system of equations

3.14 $$\begin{aligned} {\left\{ \begin{array}{ll} \frac{1}{N^\gamma }\Big (D_1\frac{E_{B}-E_A}{3}+ D_2\frac{E_A-E_{C}}{3}\Big )+D_1v=0 \\ \frac{1}{N^\gamma }\Big (D_1\frac{E_{B}-E_{C}}{3}- D_2\frac{E_{B}-E_A}{3}\Big )+D_2v=0. \end{array}\right. } \end{aligned}$$

As the system is overdetermined, we fix $D_1=1$. Then, we obtain two solutions, which, in the notation of Sect. 2.2, are given by

- $D_2=D_2^+=\frac{c_-}{E_A-E_C}$ for $c_-$ given in (2.15) with $v_+=\frac{\delta }{2N^\gamma }$ and $\delta$ defined according to (2.16).
- $D_2=D_2^-=\frac{c_+}{E_A-E_C}$ for $c_+$ given in (2.15) with $v_-=-\frac{\delta }{2N^\gamma }$ and $\delta$ as above.

We are therefore led to consider the fields3.15whose corresponding martingales can be written as3.16where3.17and  contains the terms that vanish in $L^2({\mathbb {P}}_{\nu _\rho })$ as $N\rightarrow +\infty$. We now investigate the different cases that arise by varying the relation among the constants $E_\alpha$ for $\alpha \in \{A,B,C\}$.

### Case (I): $E_{A}-E_{C}=E_{B}-E_{C}=E$

Under the hypothesis on the rates, the system in (3.14) with $D_1=1$, is solved by the following values of the parameters

- $$D_2=D_2^+=-1$$, $$v=v_+=\frac{E}{3N^\gamma }$$
- $$D_2=D_2^-=1$$, $$v=v_-=-\frac{E}{3N^\gamma }$$

which give the fields in (2.12). From (3.16), the martingales read3.18whereand, by (3.17)3.19and  is a term whose $L^2(\mathbb P_{\nu _\rho })$-norm vanishes as $N\rightarrow \infty$.

Observe that this choice of the fluctuation fields matches that of Appendix A.1. Moreover the prediction is that, in the strong asymmetric regime (i.e. for $\gamma =0$), the first field should have diffusive behaviour while the second should have KPZ behaviour. To see this (at least in the weakly asymmetric case), it remains to analyse the term .

#### The field 

From (3.17), we have3.20From the second-order Boltzmann-Gibbs Principle, namely Theorem 6.2, for $\gamma >1/2$ the term  vanishes in $L^2({\mathbb {P}}_{\nu _{\rho }})$ as $N\rightarrow \infty$. For $\gamma =1/2$ instead, it converges to a non-trivial limit. To see what this limit is, note that, for $x\in \mathbb {Z}$, $\alpha , \beta \in \{A,B\}$ and $\varepsilon >0$, Theorem 6.2 allows us to replace ${{\bar{\xi }}}^{\alpha }_x$ and ${{\bar{\xi }}}^{\beta }_{x+1}$ with $\overleftarrow{\xi }^{\varepsilon N,\alpha }_{x}$ and $\overrightarrow{\xi }^{\varepsilon N,\beta }_{x}$ respectively, which are the centred averages of ${{\bar{\xi }}}^{\alpha }_x$ and ${{\bar{\xi }}}^{\beta }_{x+1}$ on a box of size $\varepsilon N$ to the left and to the right of *x*, see (6.1). Here and in what follows $\epsilon N$ should be interpreted as $\lfloor \epsilon N\rfloor$. Hence, modulo terms whose $L^2({\mathbb {P}}_{\nu _{\rho }})$-norm vanish as $N\rightarrow \infty$,  becomes3.21Let us now define for $u,v\in {\mathbb {T}}$ the functions3.22Note that $\overrightarrow{i_\varepsilon }(u)=\overrightarrow{i_\varepsilon }(u)(\cdot )=\overrightarrow{i_\varepsilon }(0)(\cdot -u)$, $\overleftarrow{i_\varepsilon }(u)=\overleftarrow{i_\varepsilon }(u)(\cdot )=\overleftarrow{i_\varepsilon }(0)(\cdot -u)$ and that for any $d\in {\mathbb {R}}$, we have that $T_d \overrightarrow{i_\varepsilon }(u-d)(v)=\overrightarrow{i_\varepsilon }(u)(v)$ and $T_d\overleftarrow{i_\varepsilon }(u-d)(v)=\overleftarrow{i_\varepsilon }(u)(v)$.

Since  has a velocity $v_-$ in its definition, we get that3.23The same identity above holds replacing $\overrightarrow{i_\varepsilon }$ by $\overleftarrow{i_\varepsilon }$ and $\overrightarrow{\xi }^{\alpha ,\varepsilon N}_\cdot$ by $\overleftarrow{\xi }^{\alpha ,\varepsilon N}_\cdot$, $\alpha \in \{A,B\}$.

From the last identities we rewrite (3.21) as3.24In conclusion, by the change of variables $z=x+\frac{E}{3} N^{3/2}s$, we write  as3.25This last expression suggests that, in the limit $N\rightarrow \infty$ and $\varepsilon \rightarrow 0$, the martingale  converges to that in (2.22) with $\lambda =E$. Hence,  displays KPZ behaviour when $\gamma =1/2$.

#### The field 

From (3.17), we have3.26Once again, for $\gamma >1/2$,  vanishes in view of the second-order Boltzmann-Gibbs principle and for $\gamma >1/2$ this field has diffusive behaviour.

For $\gamma =1/2$, we will need instead the second version of the principle, Theorem 6.4. To see how to apply it, we introduce the centred empirical measure ${{\bar{\pi }}}^{N,\alpha }_s$, $\alpha \in \{A,B,C\}$, which is defined as

3.27 $$\begin{aligned} {{\bar{\pi }}}^{N,\alpha }_s(du)=\frac{1}{N}\sum _{x\in {\mathbb {T}}_N}[\xi ^\alpha _x(s)-\rho _\alpha ]\delta _{\frac{x}{N}}(du). \end{aligned}$$

Observe that this is nothing but the density field  but in a different scaling regime.

With this definition at hand, we have

3.28 $$\begin{aligned} \begin{aligned}&\langle {{\bar{\pi }}}^{N,\alpha }_s,\overleftarrow{\rho _{\varepsilon ,\delta }}(\cdot -\tfrac{x}{N})\rangle =\frac{1}{N}\sum _{y}\overleftarrow{\rho _{\varepsilon ,\delta }}\Big (\frac{y-x}{N}\Big ){{\bar{\xi }}}^\alpha _y(s),\quad \text {and}\\  &\quad \langle {{\bar{\pi }}}^{N,\alpha }_s,\overrightarrow{\rho _{\varepsilon ,\delta }}(\cdot -\tfrac{x}{N})\rangle =\frac{1}{{N}}\sum _{y }\overrightarrow{\rho _{\varepsilon ,\delta }}\Big (\frac{y-x}{N}\Big ){{\bar{\xi }}}^\alpha _y(s) \end{aligned} \end{aligned}$$

where $\overleftarrow{\rho _{\varepsilon ,\delta }}$ and $\overrightarrow{\rho _{\varepsilon ,\delta }}$ are smooth functions with compact support in $[0,\varepsilon ]$ and $[-\varepsilon ,0]$ and are defined in the text above (6.12). For $x\in \mathbb {Z}$, $\alpha , \beta \in \{A,B\}$ and $\varepsilon>\delta >0$, Theorem 6.4 allows us to replace ${{\bar{\xi }}}^{\alpha }_{x}{{\bar{\xi }}}^{\beta }_{x+1}$ by

$$\begin{aligned} \langle {{\bar{\pi }}}^{N,\alpha }_s,\overleftarrow{\rho _{\varepsilon ,\delta }}(\cdot -\tfrac{x}{N})\rangle \langle {{\bar{\pi }}}^{N,\beta }_s,\overrightarrow{\rho _{\varepsilon ,\delta }}(\cdot -\tfrac{x}{N})\rangle , \end{aligned}$$

at a price whose second moment is $O(\varepsilon +\delta /\varepsilon ^2)$, and thus vanishes as $\delta \rightarrow 0$ and $\varepsilon \rightarrow 0$. Therefore, modulo negligible terms,  can be written asTo identify the density fields in the previous display, let us focus on the first summand, and note that by a change of variableswhere the last passage follows arguing as in (3.23) and using the fact that fields are defined with opposite velocities. Applying the same strategy for the second summand, we get3.29

##### Remark 3.2

The reason why we need to consider the novel version of the second-order Boltzmann-Gibbs principle is to control terms as those in (3.29), i.e. terms displaying products of fields living in moving frames with different velocities. Indeed, in order to determine their limiting behaviour we will need to invoke Theorem 5.1 which only applies if the fields are tested against sufficiently smooth functions (and this would not be the case if $\overrightarrow{\rho _{\varepsilon ,\delta }}$ and $\overleftarrow{\rho _{\varepsilon ,\delta }}$ were replaced by $\overrightarrow{i_\varepsilon }$ and $\overleftarrow{i_\varepsilon }$).

In conclusion, we obtain3.30Now, Theorem 5.1 with $\varphi _1,\varphi _2\in \{\overrightarrow{\rho _{\varepsilon ,\delta }},\overleftarrow{\rho _{\varepsilon ,\delta }}\},$ suggests that, as $N\rightarrow \infty$, the last term only contributes via the limiting mass of , so that the martingale  should converge to that in (2.22) with $\lambda =0$. Therefore this field has a diffusive behaviour (for any $\gamma >1/2$).

##### Remark 3.3

We note that in the last line of (3.30), the field ${\mathscr {Z}}_t^{N,-}$ is being observed in a time frame that does not coincide with the one in which it converges, and this corresponds to be taking ${\mathscr {Z}}_t^{N,-}$ in a much longer time scale. In other words, the velocity makes it so that, in a time interval of order $t/\varepsilon ^2$ (i.e. the diffusive scaling we are considering), the field “sees” the whole torus multiple times so that we can replace the approximate indicator $\overrightarrow{ \rho _{\varepsilon ,\delta }}$ of a local box of size $\varepsilon N$ to an indicator of the box of order *N*. Since the total number of particles is conserved by the dynamics, this results in an additional massive term in the limit. It is thanks to Theorem 5.1, that we can make this argument rigorous.

### Case (II): $E_{B}-E_{A}=E_{C}-E_{A}=E$

As previously observed in Remark 2.4, this case can be obtained from case **(I)** by a simple trasformation of the fields, so we omit the details and present only the final formulation of the martingale problems. Recall (2.13), i.e. For the first field, when $\gamma =1/2$, we have for , So we expect that any limit point  of the sequence  satisfies (2.22) with $\lambda _-=E$ and so in this case, i.e. $\gamma =1/2$, the field has KPZ behaviour. As before, for $\gamma >1/2$, any crossed term in the occupation variables vanishes in the $N\rightarrow \infty$ limit and we observe diffusive behaviour.

For the second field, we proceed exactly as in Sect. 3.2.2 and get to the following expression for the associated martingale3.31Once again, Theorem 5.1 implies that the last term only contributes via the limiting mass of , which suggests that (3.31) will converge to (2.22) with $\lambda =0$.

### Case (III): $E_{C}-E_{A}\ne E_{C}-E_{B}\ne 0$

In this case, we consider the generic fields given in (3.15). Let us start with  and then we analyze , keeping in mind that both modes are predicted to have KPZ behaviour. To make the presentation simpler, in this subsection we make the choice $E_C=0$, as one can recover the general case by replacing every instance of $E_A$ and $E_B$ below with $E_A-E_C$ and $E_B-E_C$, respectively.

#### The field 

Plugging the values $D_2=D_2^-=c_+/E_A$, $D_1=1$ and $v=v_-=-\tfrac{\delta }{2N^\gamma }$, for $\delta$ in (2.16), into the formula (3.11) and ignoring both the terms that are negligible in the limit as $N\rightarrow \infty$ and those killed with the choice of constants and velocities, we see that for any  we have3.32The second order Boltzmann-Gibbs principle says that the last three lines in the last display vanish as $N\rightarrow \infty$ for $\gamma >1/2$, and in this case the field has diffusive behaviour. When $\gamma =1/2$, in order to close the equations in terms of the fields  and  we first note that the last three lines in last display can be written as

3.33 $$\begin{aligned} \begin{aligned}&\int _0^t \text {d}s \sum _{x\in {\mathbb {T}}_N}\nabla _N T_{-\frac{\delta }{2} N^{3/2}s}f\left( \tfrac{x}{N}\right) \Big \{E_A{{\bar{\xi }}}^A_{x}(s){{\bar{\xi }}}^A_{x+1}(s)\\  &\quad + \frac{E_A+\tfrac{3}{2}\delta }{2}\Big ({\bar{\xi }}^{A}_{x}(s){\bar{\xi }}^{B}_{x+1}(s)+{\bar{\xi }}^{B}_{x}(s){\bar{\xi }}^{A}_{x+1}(s)\Big )\\&\quad +\frac{c_+E_{B}}{E_A}{{\bar{\xi }}}^{B}_{x}(s){{\bar{\xi }}}^{B}_{x+1}(s)\Big \}. \end{aligned} \end{aligned}$$

Let us look at the term in parenthesis. Note that

3.34 $$\begin{aligned} \begin{aligned}&E_A{{\bar{\xi }}}^A_{x}{{\bar{\xi }}}^A_{x+1}+ \frac{E_A+\tfrac{3}{2}\delta }{2}\Big ({\bar{\xi }}^{A}_{x}{\bar{\xi }}^{B}_{x+1}+{\bar{\xi }}^{B}_{x}{\bar{\xi }}^{A}_{x+1}\Big )+\frac{c_+E_{B}}{E_A}{{\bar{\xi }}}^{B}_{x}{{\bar{\xi }}}^{B}_{x+1}\\&\quad ={\mathfrak {c}}_1 \Big ({{\bar{\xi }}}_x^A+\frac{c_+}{E_A}{{\bar{\xi }}}_{x}^{B}\Big ) \Big ( {{\bar{\xi }}}_{x+1}^A+\frac{c_+}{E_A}{{\bar{\xi }}}_{x+1}^{B}\Big )\\&\qquad +{\mathfrak {c}}_2 \Big ( {{\bar{\xi }}}_x^A+\frac{c_+}{E_A}{{\bar{\xi }}}_{x}^{B}\Big ) \Big ( {{\bar{\xi }}}_{x+1}^A+\frac{c_-}{E_A}{{\bar{\xi }}}_{x+1}^{B}\Big )\\&\qquad + {\mathfrak {c}}_3 \Big ( {{\bar{\xi }}}_x^A+\frac{c_-}{E_A}{{\bar{\xi }}}_{x}^{B}\Big )\Big ( {{\bar{\xi }}}_{x+1}^A+\frac{c_+}{E_A}{{\bar{\xi }}}_{x+1}^{B}\Big ) \end{aligned} \end{aligned}$$

for $${\mathfrak {c}}_2={\mathfrak {c}}_3=(E_A-{\mathfrak {c}}_1)/2$$ and

$$\begin{aligned}\begin{aligned} {\mathfrak {c}}_1= \frac{E_A}{3\delta }(2E_{B}-E_{A}+\tfrac{3}{2}\delta )=\frac{E_A}{3\delta }(E_B-c_-) \end{aligned} \end{aligned}$$

where $c_\pm$ and $\delta$ are defined in (2.15) and (2.16). From this we conclude that (3.33) can be rewritten as

$$\begin{aligned} \begin{aligned}&\int _0^t \text {d}s \sum _{x\in {\mathbb {T}}_N}\nabla _N T_{v_- N^2s}f\left( \tfrac{x}{N}\right) \Big \{\frac{E_A}{3\delta }(E_B-c_-) \Big ({{\bar{\xi }}}_x^A+\frac{c_+}{E_A}{{\bar{\xi }}}_{x}^{B}\Big ) \Big ( {{\bar{\xi }}}_{x+1}^A+\frac{c_+}{E_A}{{\bar{\xi }}}_{x+1}^{B}\Big )\\&\quad + E_A\Big (\frac{1}{2}-\frac{E_B-c_-}{6\delta }\Big ) \Big ( {{\bar{\xi }}}_x^A+\frac{c_+}{E_A}{{\bar{\xi }}}_{x}^{B}\Big ) \Big ( {{\bar{\xi }}}_{x+1}^A+\frac{c_-}{E_A}{{\bar{\xi }}}_{x+1}^{B}\Big ) \\&\quad + E_A\Big (\frac{1}{2}-\frac{E_B-c_-}{6\delta }\Big ) \Big ( {{\bar{\xi }}}_x^A+\frac{c_-}{E_A}{{\bar{\xi }}}_{x}^{B}\Big )\Big ( {{\bar{\xi }}}_{x+1}^A+\frac{c_+}{E_A}{{\bar{\xi }}}_{x+1}^{B}\Big ) \Big \}. \end{aligned} \end{aligned}$$

Now, in the first line we apply the standard version of the second order Boltzmann–Gibbs principle, and in the second and third we use the new formulation proposed in Theorem 6.4, so that overall the previous display equals As in the previous cases, the terms in the last two lines of the last display only produce linear functionals in the limit $N\rightarrow \infty$ and the only one that survives is the first, so that  has KPZ behaviour (for $\gamma =1/2$).

#### The field 

Dynkin’s formula for the field  is identical to the expression in (3.32), with $c_-$ instead of $c_+$ and $\delta$ replaced by $-\delta$. For $\gamma >1/2$ we know that terms in  vanish as $N\rightarrow \infty$ while for $\gamma =1/2$ they have a non-trivial limit. As above, we want to write the current in terms of the fluctuation fields  and . To do so, we solve the same linear system as in (3.34) but in which we exchange $c_+$ and $c_-$. This leads us to3.35Arguing exactly as above, we ultimately get3.36which suggests that also this field has KPZ behaviour for $\gamma =1/2$.

## Proof of Theorem 2.9

In this section, we prove the main result of the paper. We will first show tightness of the fluctuation fields and then uniquely characterise the limit points by verifying they satisfy either Definition 2.5 or 2.6.

### Tightness

In this section we prove not only tightness of the sequences of processes  with respect to the uniform topology of , in each of the cases **(I)**-**(III)** above, but further show some properties the fluctuation fields enjoy and that will be necessary to control terms containing their cross product. The main result of the section is the following proposition.

#### Proposition 4.1

In any of the cases **(I)-(III)** in Sect. 2.2, the sequences of processes  are tight in .

Furthermore, there exists a constant $C>0$ such that for any  and $N\in \mathbb {N}$, the following bounds hold4.14.2where $\Vert \cdot \Vert _2$ denotes the usual $L^2(\mathbb {T})$-norm.

#### Proof

By Mitoma’s criterion [28], tightness for  follows upon showing that, for any , the sequences  are tight.

Now, notice that, in each of the cases **(I)**-**(III)** of the previous section, the fields  can be written, in their most general form, as in (3.11) where $a,b,D_1,D_2$ and *v* are chosen in such a way that the fourth, fifth and eighth lines sum up to 0. In other words, we look at the generic field  in (3.1), which is given by4.3where  is the martingale whose quadratic variation is given in (3.3),4.44.5for suitable choices of the constants ${\mathfrak {C}}_i$, $i=1,2,3$, and  consists of the sixth and seventh lines in (3.11), so that its second moment goes to 0 as $N\rightarrow \infty$. We will separately analyse the terms on the right-hand side of (4.3) and show that each of them is tight and satisfies (4.1) and (4.2).

The process  can be explicitly written as4.6We consider its increment and apply the Cauchy–Schwarz inequality to the time integral thus obtaining4.7where we used the fact that the product measure $\nu _\rho$ is stationary and that the expected value in the second to last line is bounded by a constant.

The previous bound guarantees, on the one hand that (4.2) holds, and on the other, by the Kolmogorov criterion, that the sequence of processes  is tight with respect to the uniform topology of ${\mathscr {C}}([0,T],\mathbb {R})$, satisfies (4.1) and any limit point has $\alpha$-Hölder-continuous trajectories for any $\alpha <1/2$,

We now turn to the sequence of quadratic terms  in (4.5). It suffices to prove tightness for each of the summands, which are all of the form4.8where $\alpha ,\beta \in \{A,B\}$. We present the proof only in the case $\alpha =\beta$, being it the hardest since it involves an extra bound on the variance of the occupation variables. As $\alpha =\beta$ and $\alpha$ will be fixed throughout, we shorten the notation by omitting the corresponding index from .

In order to estimate the second moment of the increments of , we will use the second order Boltzmann–Gibbs principle, which allows to replace the single-species degree-two term ${{\bar{\xi }}}^\alpha _x{{\bar{\xi }}}^\alpha _{x+1}$ with the local function of the configuration $\overleftarrow{\xi }^{\alpha ,L}_x\overrightarrow{\xi }^{\alpha ,L}_x$, for some *L* to be determined. If we sum and subtract in the integral in (4.8) the term $\overleftarrow{\xi }^{\alpha ,L}_x\overrightarrow{\xi }^{\alpha ,L}_x(sN^a)$ and use the convex inequality $(x+y)^2\le 2x^2+2y^2$ then, for any $0\le s<t\le T$ it holds4.9Theorem 6.2 implies that the first term is bounded above by

4.10 $$\begin{aligned} \begin{aligned} N^{1-2\gamma }\frac{L}{N}\int _s^t\text {d}r\frac{1}{N}&\sum _{x\in \mathbb T_N}\left[ \nabla _NT_{v N^2r}f\left( \tfrac{x}{N}\right) \right] ^2\lesssim N^{1-2\gamma }\frac{(t-s)L}{N}\Vert \nabla f\Vert ^2_{2}, \end{aligned} \end{aligned}$$

while, from the Cauchy–Schwarz inequality, the second term can be bounded above by4.11Note that for $|x-y|>L$ the variables $\overrightarrow{\xi }^{\alpha ,L}_x(r)$ and $\overrightarrow{\xi }^{\alpha ,L}_y(r)$ are uncorrelated and mean zero, which implies that we can restrict the double sum to $|x-y|\le L$. We then apply again the convex inequality $2xy\le x^2+y^2$, to estimate the previous display by a constant times4.12Putting together (4.10) and (4.11), and choosing $L=\sqrt{(t-s)} N^{2\gamma }$, we get4.13Since the previous computations can be derived for any $\alpha , \beta \in \{A,B\}$, we conclude that4.14We can now argue as for  to conclude that tightness, (4.1) and (4.2) hold for .

At last, we consider the sequence of martingales . We will prove a much stronger statement, namely that the sequence indeed converges (so that in particular it is tight) and that the limit is a Brownian motion with an explicit covariance. To do so, we exploit [24, Theorem VIII, 3.12] that we state below for the reader’s convenience.

#### Theorem 4.2

Let $\{M^N\}_{N \in {\mathbb {N}}}$ be a sequence of martingales belonging to the space $D ([0,T]; {\mathbb {R}})$ and denote by $\langle M^N\rangle$ the quadratic variation of $M^N$, for any $N\in {\mathbb {N}}$. Let $c:[0,T]\rightarrow [0,\infty )$ be a deterministic continuous function. Assume that

1. For any $N\in {\mathbb {N}}$, the quadratic variation process $\langle M^N\rangle _t$ has continuous trajectories almost surely,
2. The following limit holds
  4.15 $$\begin{aligned} \lim _{N \rightarrow \infty }{\mathbb {E}}_{\nu _{\rho }}\Big [\sup _{0\le s \le T}\Big |M_s^N-M_{s^-}^N\Big |\Big ]=0, \end{aligned}$$
3. For any $t \in [0,T]$ the sequence of random variables $\{\langle M^N\rangle _t\}_{N> 1}$ converges in probability to *c*(*t*).

Then, the sequence $\{M^N\}_{N}$ converges in law in $D([0,T]; \mathbb {R})$, as *N* goes to infinity, to a mean zero Gaussian process *M* which is a martingale on [0, *T*] with continuous trajectories and whose quadratic variation is given by *c*.

Point 1. in the previous statement is trivially satisfied by our sequence  as the quadratic variation in (3.3) is clearly continuous. Now, for point 2., note that4.16To bound the right-hand side, we consider a particle exchange happening at time *s* on the bond $\{x,x+1\}$. If at time $s^-$, we observe the pair (*A*, *B*), then at time *s* we will have the pair (*B*, *A*). Therefore, at site *x*,

$$\begin{aligned} \Big (D_1{\bar{\xi }}^{A}_x(s)+D_2{\bar{\xi }}^{B}_x(s)\Big )-\Big (D_1{\bar{\xi }}^{A}_x(s^-)+D_2{\bar{\xi }}^{B}_x(s^-)\Big )=D_2-D_1, \end{aligned}$$

while at site $x+1$,

$$\begin{aligned} \Big (D_1{\bar{\xi }}^{A}_{x+1}(s)+D_2{\bar{\xi }}^{B}_{x+1}(s)\Big )-\Big (D_1{\bar{\xi }}^{A}_{x+1}(s^-)+D_2{\bar{\xi }}^{B}_{x+1}(s^-)\Big )=D_1-D_2. \end{aligned}$$

From this we getand, since the right-hand side does not depend on *s*, point 2. trivially follows. For point 3., we first state a crucial lemma whose proof is provided at the end of the section.

#### Lemma 4.3

For any , it holds

The previous statement, together with Lemma 3.1 completes the verification of the assumptions of Theorem 4.2. Hence, the sequence  is tight and converges in law to the Gaussian process whose quadratic variation is given by the right hand side of (3.4).

It remains to prove the validity of (4.1) and (4.2) for . For the first, by Burkholder–Davis–Gundy inequality, (3.4) and (4.16) we obtain4.17For the latter instead, recalling the expression of the quadratic variation of the martingale given in (3.3), we see that for any $0\le s<t\le T$, we have4.18To summarise, each of the terms , ,  and  is tight, which implies tightness for . Moreover, (4.7), (4.18), and (4.14) give (4.2), while (4.1) follows by (4.17) and the uniform (in *N*) continuity of , . $\square$

#### Proof of Lemma 4.3

Note that the expectation in the statement of the lemma is equal to

$$\begin{aligned} \begin{aligned}&{\mathbb {E}}_{\nu _\rho }\bigg [\bigg (\int _0^t \text {d}s\frac{1}{N}\sum _x (\nabla _N T_{v N^2s}f(\tfrac{x}{N}))^2\\&\quad \times \Big [c_x(\eta )\Big \{\Big (D_1\xi ^{A}_{x+1}+D_2\xi ^{B}_{x+1})-(D_1\xi ^{A}_{x}+D_2\xi ^{B}_{x})\Big \}^2\\&\quad -\frac{4}{9}(D_1^2+D_2^2-D_1D_2)\Big ]\bigg )^2\bigg ]. \end{aligned} \end{aligned}$$

From the computations in the proof of Lemma 3.1 we know that

4.19 $$\begin{aligned} \begin{aligned}&c_x(\eta )\Big \{\Big (D_1\xi ^{A}_{x+1}+D_2\xi ^{B}_{x+1})-(D_1\xi ^{A}_{x}+D_2\xi ^{B}_{x})\Big \}^2-\frac{4}{9}(D_1^2+D_2^2-D_1D_2)\\&\quad =D_1^2\Big \{\sum _{\alpha }c^{\alpha , A}\xi ^{\alpha }_{x}\xi ^{A}_{x+1}+\sum _\beta c^{A, \beta }\xi ^{A}_{x}\xi ^{\beta }_{x+1}-\frac{4}{9}\Big \}\\&\qquad + D_2^2\Big \{\sum _{\alpha }c^{\alpha , B}\xi ^{\alpha }_{x}\xi ^{B}_{x+1}+\sum _\beta c^{B,\beta }\xi ^{B}_{x}\xi ^{\beta }_{x+1}-\frac{4}{9}\Big \}\\  &\qquad -2D_1D_2\Big \{c^{B,A}\xi ^B_x\xi ^A_{x+1}+c^{A,B}\xi _x^A\xi _{x+1}^B-\frac{2}{9}\Big \}. \end{aligned} \end{aligned}$$

The proof of the lemma is complete once we show that the second moment of the time integral of each of the previous summands goes to 0 as $N\rightarrow \infty$. As they can all be analysed similarly, we limit ourselves to treat the first. By the Cauchy-Schwarz inequality, we have

4.20 $$\begin{aligned} \begin{aligned}&{\mathbb {E}}_{\nu _\rho }\bigg [\bigg (\int _0^t \text {d}s\frac{1}{N}\sum _x (\nabla _N T_{v N^2s}f(\tfrac{x}{N}))^2\Big \{\sum _{\alpha }c^{\alpha , A}\xi ^{\alpha }_{x}\xi ^{A}_{x+1}\!+\!\sum _\beta c^{A, \beta }\xi ^{A}_{x}\xi ^{\beta }_{x+1}-\frac{4}{9}\Big \}\bigg )^2\bigg ]\\&\le \frac{t^2}{N^2}\sum _{x,y}\nabla _N f(\tfrac{x}{N})^2\nabla _N f(\tfrac{y}{N})^2\mathbb E_{\nu _\rho }\bigg [\Big \{\sum _{\alpha }c^{\alpha , A}\xi ^{\alpha }_{x}\xi ^{A}_{x+1}+\sum _\beta c^{A, \beta }\xi ^{A}_{x}\xi ^{\beta }_{x+1}-\frac{4}{9}\Big \}\\&\qquad \times \Big \{\sum _{\alpha }c^{\alpha , A}\xi ^{\alpha }_{y}\xi ^{A}_{y+1}+\sum _\beta c^{A, \beta }\xi ^{A}_{y}\xi ^{\beta }_{y+1}-\frac{4}{9}\Big \}\bigg ]. \end{aligned}\nonumber \\ \end{aligned}$$

Note now that for $|x-y|>1$ the variables $\xi ^{\alpha }_{x}\xi ^{\beta }_{x+1}$ and $\xi ^{\alpha '}_{y}\xi ^{\beta '}_{y+1}$ are independent for any value of $\alpha ,\alpha ',\beta ,\beta '\in \{A,B\}$. Since they are also centred, the expectation at the right hand side is 0. When instead $x\in \{y,y+1\}$ the expectation can be easily seen to be bounded so that overall (4.20) is of order $N^{-1}$ and therefore vanishes in the limit. $\square$

### Characterisation of the limit points

From the results in the previous section, we know that the sequences , , ,  and , the latter vanishing, are tight with respect to the uniform topology on . Prohorov’s theorem ensures the existence of a converging subsequence that, abusing notation, we will still denote by *N*. Let , ,  and  be their almost surely continuous limits.

We are left to show that  are either solutions to the Ornstein–Uhlenbeck equation or stationary energy solutions of the stochastic Burgers equation in the sense of Definitions 2.6 and 2.5. Once this is proven, since any of these solutions is unique by Theorem 2.7, the convergence of the whole sequence follows.

We begin with item (i) in Definition 2.5. Note that, by computing the characteristic function at any time *t* of the field  we obtain that  converges in distribution to a spatial white noise of variance

4.21 $$\begin{aligned} \text {Var}(D_1\xi _x^A+D_2\xi _x^{B})=\frac{2}{9}(D_1^2+D_2^2-D_1D_2), \end{aligned}$$

where the previous equality can be verified by arguing as in the proof of Lemma 3.1. Above for a random variable *X*, we denoted by $\text {Var}(X)$ its variance with respect to $\nu _\rho$.

Now, we turn to items (ii)-(iii). The initial field  can be treated as in the previous point. The martingale terms  were thoroughly analysed in the previous section, where it was shown that  is a mean-zero Gaussian process with quadratic variation given by the right-hand side of (3.4). For the linear term , recall thatSince  converges in the space , the limit must be such that for any At this point, it remains to study the quadratic term , which is the most delicate. Recall (3.17).

In all the cases that we distinguished in Sect. 3, from the second-order Boltzmann-Gibbs principles, Theorems 6.2 and 6.4, (3.17) vanishes in $L^2(\mathbb P_{\nu _\rho })$ if $\gamma >1/2$, while for $\gamma =1/2$ it can be written as the sum of terms that can be either of the form4.22where $\lambda _\pm$ is a constant and $\iota ^i_\varepsilon \in \{\overleftarrow{\iota _\varepsilon }, \overrightarrow{\iota _\varepsilon }\}$, $i=1,2$, or4.23for some constants ${\mathfrak {A}}^\pm$, where $\rho _{\varepsilon ,\delta }^i \in \{\overleftarrow{\rho _{\varepsilon ,\delta }}, \overrightarrow{\rho _{\varepsilon ,\delta }}\}$, $i=1,2$, see above (6.12) for the definition of these functions. To control this latter integral we would like to apply Theorem 5.1 but, while condition 2 clearly holds with $\alpha =1/2$ as can be seen by a simple application of Cauchy-Schwarz inequality together with (4.1) and (4.2), condition 1. is violated for the points $k_1=k\in \mathbb {Z}{\setminus }\{0\}$ and $k_2=0$. Indeed, for these values of $k_1,k_2$ we have $k_1+k_2=k\ne 0$, $\widehat{\rho ^1_{\varepsilon ,\delta }}(k_1)\widehat{\rho ^2_{\varepsilon ,\delta }}(0)\ne 0$ but $v_1^Nk_1+v_2^N k_2= 0 \times k +2v_\pm N^{2} \times 0=0$. This is the place at which the linear transport term in (2.23) comes about. To see this, note first that, by definition, $\widehat{\rho ^2_{\varepsilon ,\delta }}(0)=\int \rho ^2_{\varepsilon ,\delta }(u)\text {d}u=1$ and4.24where we used that the total mass is conserved and time-independent, and denoted it by . Hence, we rewrite (4.23) as4.25We can now apply Theorem 5.1 which ensures that, as $\varepsilon >0$ and $\delta$ are fixed, the first summand vanishes in the limit $N\rightarrow \infty$. The second instead is the product of two terms which are tight (it is *not* the product of fields anymore!) and therefore it converges, in the limit $N\rightarrow \infty$ first, then $\delta \rightarrow 0$ and at last $\varepsilon \rightarrow 0$, to4.26We turn to (4.22), for which, by tightness of , we get4.27The process at the left-hand side coincides with that in (2.19). Indeed, even if neither $\overleftarrow{\iota _\varepsilon }$ nor $\overrightarrow{\iota _\varepsilon }$ belong to , they can be approximated by elements in the latter space (see also the footnote before (2.19)). For further details, we refer the reader to [18, Section 5.3]. Now, by computations similar to those performed in the proof of (4.14), it is not hard to see that for any $0<\delta \le \varepsilon <1$, we haveso that, by (4.27) and taking the limit in *N*, we obtain4.28which, on the one hand guarantees that  has unique limit in $\varepsilon \rightarrow 0$, and on the other provides the energy estimate (2.20). Hence, both (ii) and (iii) of Definition 2.5 hold.

At last, we note that all the arguments above hold *mutatis mutandis* for the reversed process  whose dynamics is generated by the adjoint operator $L_N^*$, so that also item (iv) is satisfied.

At last, we prove that  and  are independent, which amounts to verify that for all ,  and  are independent. This in turn follows once we show that the cross-variation  By polarization, for any $N\ge 1$, we haveand we will provewhich completes the proof.

A simple computation similar to that in (3.3) shows that4.29Now, the first two terms on the right-hand side equal  and , respectively, so that we only need to show that the variance of the last vanishes as $N\rightarrow +\infty$. To this end, we first note that its mean is zero since

4.30 $$\begin{aligned} \begin{aligned}&{\mathbb {E}}_{\nu _\rho }\Big [\big ( \xi ^{A}_{x+1}-\xi ^{A}_{x}+D_2^+ (\xi ^{B}_{x+1}-\xi ^{B}_{x})\big )\big ( \xi ^{A}_{x+1}-\xi ^{A}_{x}+D_2^- (\xi ^{B}_{x+1}-\xi ^{B}_{x})\big )\Big ]\\  &\quad =\frac{2}{9} (2-(D_2^++D_2^-)+2D_2^+D_2^-)=0 \end{aligned} \end{aligned}$$

where the last equality holds provided $D_2^+, D_2^-$ are chosen as in cases **(I-III)**. Here, we are crucially relying on the choice of constants dictated by mode coupling theory. Turning to the second moment, we perform computations similar to those in the proof of Lemma 4.3. By the Cauchy-Schwarz inequality, for $a=2$, we have

$$\begin{aligned} \begin{aligned}&{\mathbb {E}}_{\nu _\rho }\Big [\Big (N^{a-3}\int _0^t \sum _x c_x(\eta )\nabla _N T_{v_+ N^2s}f(\tfrac{x}{N})\nabla _N T_{v_- N^2s}g(\tfrac{x}{N})\times \\&\quad \times \big [ \xi ^{A}_{x+1}-\xi ^{A}_{x}+D_2^+ (\xi ^{B}_{x+1}-\xi ^{B}_{x})\big ]\big [ \xi ^{A}_{x+1}-\xi ^{A}_{x}+D_2^- (\xi ^{B}_{x+1}-\xi ^{B}_{x})\big ]\text {d}s\Big )^2\Big ]\\&\le \frac{t}{N^2}\int _0^t\text {d}s \sum _{x,y} \nabla _N T_{v_+ N^2s}f(\tfrac{x}{N})\nabla _N T_{v_- N^2s}g(\tfrac{x}{N})\nabla _N T_{v_+ N^2s}f(\tfrac{y}{N})\nabla _N T_{v_- N^2s}g(\tfrac{y}{N})\times \\&\quad \times {\mathbb {E}}_{\nu _\rho }\Big [c_x(\eta )c_y(\eta )\big ( \xi ^{A}_{x+1}-\xi ^{A}_{x}+D_2^+ (\xi ^{B}_{x+1}-\xi ^{B}_{x})\big )\big ( \xi ^{A}_{x+1}-\xi ^{A}_{x}+D_2^- (\xi ^{B}_{x+1}-\xi ^{B}_{x})\big )\times \\&\quad \times \big ( \xi ^{A}_{y+1}-\xi ^{A}_{y}+D_2^+ (\xi ^{B}_{y+1}-\xi ^{B}_{y})\big )\big ( \xi ^{A}_{y+1}-\xi ^{A}_{y}+D_2^- (\xi ^{B}_{y+1}-\xi ^{B}_{y})\big )\Big ]\\ \end{aligned} \end{aligned}$$

and, arguing as in (4.19)-(4.20), since the variables are independent and centred, the terms in the last sum that survive are only those for which $|x-y|\le 1$. Therefore, the last term vanishes as $N\rightarrow +\infty$.

Finally we prove that for every $t\in [0,T]$ the processes  and  are uncorrelated. To this end we note that for any *N* and for any , we have that  and  are uncorrelated i.e.4.31and this is a consequence of the fact that for any $x,y\in \mathbb T_N$

4.32 $$\begin{aligned} \mathbb E_{\nu _\rho }[({{\bar{\xi }}}^A_x+D_2^+{{\bar{\xi }}}^B_x)({{\bar{\xi }}}^A_y+D_2^-{{\bar{\xi }}}^B_y)]=0 \end{aligned}$$

for any choice of $D_2^\pm$ in all the cases **(I-III)**. This is an easy computation that is similar to the one in (4.30). Again we note that in the last result it is crucial to use the choice of the constants fixing the fields that we obtained from mode coupling theory. With this the proof of Theorem 2.9 is completed.

## Crossed fields

The goal of this section is to show that those integrals displaying the product of fluctuation fields evolving on different time frames, vanish in the large *N* limit. We state the main result in wider generality as we believe its scope goes beyond the model treated in the present work, so that the theorem below might be of independent interest.

### Theorem 5.1

Let $T>0$, $n\in \mathbb {N}$ and, for every $N\in \mathbb {N}$, let  be *n* time-dependent random fields taking values in  and defined on the same probability space. Let $\{v_i^N\}_N$, $i=1,\dots ,n$ be *n* sequences of constants and $\varphi _i$, $i=1,\dots ,n$, be smooth functions on $\mathbb {T}$.

We make the following assumptions

1. The constants $\{v_i^N\}_N$ and the functions $\varphi _i$ are such that if for $k_1,\dots ,k_n\in \mathbb {Z}$ with $\sum _{i=1}^n k_i\ne 0$, $\Pi _i{\hat{\varphi }}_i(k_i)\ne 0$, then
  5.1 $$\begin{aligned} \lim _{N\rightarrow \infty } \frac{1}{N}\left| \sum _{i=1}^n k_i v_i^N\right| =\infty , \end{aligned}$$
2. There exist $\alpha \in (0,1)$, and a constant $C>0$ such that for any , the following bounds hold uniformly in *N*5.25.3 where, for $s\in [0,T]$, we denoted by .

Then, for any $t\in [0,T]$ and , we have5.4

The proof of the previous result relies on a version of the Riemann–Lebesgue lemma which is stated in Appendix B. To be able to use it, we need to introduce some notation. Let $\phi$ be a real-valued function on $\mathbb {T}$. We define the *discrete Fourier transform* of $\phi$ as5.5where $e_k(x):=e^{2\pi \iota k x}$, for $x\in \mathbb {T}$, and $\iota :=\sqrt{-1}$, the complex unit. The usual properties of the Fourier transform hold in the discrete context as well. For the reader’s convenience, we recall them here without proof. The following inversion holds5.6For any $a\in \mathbb {R}$ the discrete Fourier transform of $\phi (a)(\cdot ):=\phi (\cdot -a)$ is given by5.7where $\lfloor a\rfloor$ denotes the integer part of *a*. Plancherel’s identity holds, i.e.5.8If $\phi \in L^2(\mathbb {T})$, then for every $k\in \mathbb {N}$5.9We are now ready to prove the result.

### Proof of Theorem 5.1

At first, we want to write the integrand in (5.4), in terms of its discrete Fourier transform. To do so, notice that, for $i=1,\dots ,n$ and any $w\in \mathbb {T}_N$, applying first (5.6) and then (5.7) to $\varphi _i(\tfrac{w}{N})(\cdot )$, we getHence, using the above and (5.8), we see that the integrand in (5.4) satisfieswhere we introduced the shorthand notation $k_{[1:n]}:=\sum _{i=1}^n k_i$ and defined $F^N$ according to5.10Now, the term we need to control is given by5.11At this point, we want to take the limit in *N* at both sides of the previous expression. Note first that by (5.2) and in view of (5.9), we have the bound5.12Now, the functions $\varphi _i$, $i=1,\dots ,n$ are infinitely differentiable (and compactly supported, as they live on $\mathbb {T}$), which implies that their Fourier transform decays faster than any polynomial. Hence, (5.12) is summable in $k_1,\dots , k_n$. Furthermore, invoking (5.9) once more, it is immediate to see that the limit in *N* of $F^N$ is given by

5.13 $$\begin{aligned} F(k_1,\dots ,k_n,k_{[1:n]}):=-2\pi \iota k_{[1:n]} {{\hat{f}}}(-k_{[1:n]})\bigg (\prod _{i=1}^n {\hat{\varphi _i}}(-k_i)\bigg ) \end{aligned}$$

which is nothing but the continuum counterpart of (5.10). By the dominated convergence theorem, we obtainNow, if $k_1,\dots ,k_n$ are such that $\sum _{i=1}^n k_i\ne 0$ or $\Pi _i{\hat{\varphi _i}}(k_i)\ne 0$, then the corresponding summand is 0 by (5.13). In all other cases, (5.1) holds and this together with the fact that the processes  satisfy condition 2, imply that Proposition B.1 is applicable and therefore the inner limit is 0, thus the proof is concluded. $\square$

## Second order Boltzmann–Gibbs principle

In this section we present two formulations of the second order Boltzmann–Gibbs principle for degree-two terms of type ${{\bar{\xi }}}^\alpha _x{{\bar{\xi }}}^{\beta }_{x+1}$, involving one or two species, i.e. for $\alpha , \beta \in \{A,B,C\}$.

### A second order Boltzmann–Gibbs principle I

The version of the Boltzmann–Gibbs principle here proposed is a multi-species generalization of Theorem 1 in [19]. The goal is to replace terms of the form ${{\bar{\xi }}}^\alpha _x{{\bar{\xi }}}^{\beta }_{x+1}$ by their respective centred averages over boxes of microscopically big (and macroscopically small) size. For this reason we introduce the left and right averages over boxes of size $\ell$ to be chosen appropriately. For $\ell \in \mathbb {N}$ and $x\in \mathbb {Z}$ we introduce the averages on boxes of size $\ell$, one to the right of *x*, one to the left:

6.1 $$\begin{aligned} \overrightarrow{\xi }^{\alpha ,\ell }_x=\frac{1}{\ell }\sum _{y=x+1}^{x+\ell }\xi ^\alpha _y,\qquad \overleftarrow{\xi }^{\alpha ,\ell }_x=\frac{1}{\ell }\sum _{y=x-\ell }^{x-1}\xi ^\alpha _y. \end{aligned}$$

#### Remark 6.1

This version of the principle is used in the analysis of the quadratic terms for the KPZ fields in cases **(I)** and **(II)**.

We first observe that our dynamics satisfies Assumptions 2.1, 2.2 and 2.3 of [19]. In fact, the invariant measure $\nu _{\rho }$ is of product form and it is such that $\int _{\Omega _N}\xi ^{\alpha }_x(\eta )\nu _{\rho }(\text {d}\eta )=\rho _\alpha$ for each *x* and each $\alpha$.

The associated Dirichlet form, defined on local functions $f\in L^2(\nu _{\rho })$, is given by

6.2 $$\begin{aligned} D_N(f)=-\int _{\Omega _N}f(\eta )L_Nf(\eta )\nu _\rho (\text {d}\eta ), \end{aligned}$$

and, using the fact that $c^{\alpha ,\beta }_x(\eta ^{x,x+1})=c^{\alpha ,\beta }_x(\eta )\frac{\nu _{\rho }(\eta ^{x,x+1})}{\nu _\rho (\eta )}$, it can be decomposed as

6.3 $$\begin{aligned} D_N(f)=\sum _{x\in {\mathbb {T}}_N}I_{x,x+1}^N(f), \end{aligned}$$

where

6.4 $$\begin{aligned} I^N_{x,x+1}(f)=\frac{N^2}{2}\int _{\Omega _N}c_x^{\alpha ,\beta }(\eta )(f(\eta ^{x,x+1})-f(\eta ))^2\nu _{\rho }(\text {d}\eta ). \end{aligned}$$

And finally we recall that the instantaneous current is given by (3.8).

Now we can state the theorem. For a function $v\in \ell ^2(\mathbb {T}_N)$ denote

$$\begin{aligned} \Vert v\Vert ^2_{2,N}:=\frac{1}{N}\sum _{x\in \mathbb {T}_N}v(x)^2<\infty . \end{aligned}$$

For a function $\phi :\Omega _N\rightarrow \mathbb {R}$, let $\Vert \phi \Vert ^2_{L^2_{\nu _\rho }}$ be the $L^2(\nu _{\rho })$-norm

$$\begin{aligned} \Vert \phi \Vert ^2_{L^2_{\nu _\rho }}=\int _{\Omega _N}\phi (\eta )^2\nu _{\rho }(\text {d}\eta ). \end{aligned}$$

#### Theorem 6.2

Let $\alpha ,\beta \in \{A,B\}$. for any $L\in \mathbb {N}$, $t>0$ and for any function $v\in \ell ^2(\mathbb {T}_N)$, we have6.5

#### Proof

Let $\ell _0\in {\mathbb {N}}$. We can split the integrand in (6.5) in the following terms

6.6 $$\begin{aligned} {{\bar{\xi }}}^{\alpha }_{x}{{\bar{\xi }}}^{\beta }_{x+1}-\overleftarrow{\xi }^{\alpha ,L}_x\overrightarrow{\xi }^{\beta ,L}_x=&{{\bar{\xi }}}^{\alpha }_{x}({{\bar{\xi }}}^{\beta }_{x+1}-\overrightarrow{\xi }^{\beta ,\ell _0}_x)\end{aligned}$$

6.7 $$\begin{aligned}&+\overrightarrow{\xi }^{\beta ,\ell _0}_x({{\bar{\xi }}}^{\alpha }_{x}-\overleftarrow{\xi }^{\alpha ,\ell _0}_x) \end{aligned}$$

6.8 $$\begin{aligned}&+\overleftarrow{\xi }^{\alpha ,\ell _0}_x(\overrightarrow{\xi }^{\beta ,\ell _0}_x-\overrightarrow{\xi }^{\beta ,L}_x) \end{aligned}$$

6.9 $$\begin{aligned}&+\overrightarrow{\xi }^{\beta ,L}_x(\overleftarrow{\xi }^{\alpha ,\ell _0}_x-\overleftarrow{\xi }^{\alpha ,L}_x)\,. \end{aligned}$$

To obtain a bound on (6.6) and (6.7) we apply the “One-block estimate”, Proposition 5 of [19]. For (6.8) and (6.9) we need a “multi-scale argument”, shown in Proposition 8 of [19].

To be concrete, applying Proposition 5 of [19] to (6.6) with $\phi (\tau _x\xi ^\alpha _0)={{\bar{\xi }}}^\alpha _x$, we obtain6.10and we do the same for (6.7) taking $\psi (\tau _x\xi ^{\beta }_0)=\overrightarrow{\xi }^{\beta ,\ell _0}_x$:6.11For (6.8) and (6.9) we apply Proposition 8 of [19] respectively:Putting together all the aforementioned estimates, we get the result. $\square$

### A second-order Boltzmann–Gibbs principle II

Here we propose an alternative version of the second order Boltzmann–Gibbs principle, where instead of replacing the occupation variable ${{\bar{\xi }}}^\alpha _x$ with left/right uniform averages over a box around *x* of size $\epsilon N$, we use smooth functions supported over $[x/N,x/N+\varepsilon ]$ or $[x/N-\varepsilon ,x/N]$.

#### Remark 6.3

This version of the principle is used in the analysis of the quadratic terms involving diffusive fields whenever we need the fields to be tested against smooth functions, i.e. whenever we need to apply Theorem 5.1.

Recall (3.22). Fix $\varepsilon >0$ and for $\delta >0$, consider smooth compactly supported functions $\overleftarrow{\rho _{\epsilon , \delta }}$ and $\overrightarrow{\rho _{\varepsilon , \delta }}$ with support in $[0,\varepsilon ]$ and $[-\varepsilon ,0]$, respectively, such that $\Vert \overleftarrow{\rho _{\varepsilon ,\delta }}\Vert _{1}=\Vert \overrightarrow{\rho _{\varepsilon ,\delta }}\Vert _{1}=1$, $\Vert \overleftarrow{\rho _{\varepsilon ,\delta }}\Vert _{2}^2\vee \Vert \overrightarrow{\rho _{\varepsilon ,\delta }}\Vert _{2}^2\le \frac{1}{\varepsilon }$, and

6.12 $$\begin{aligned} \Vert \overleftarrow{\rho _{\varepsilon , \delta }}-\overleftarrow{i_\varepsilon }(0)\Vert _{2}^2\vee \Vert \overrightarrow{\rho _{\varepsilon , \delta }}-\overrightarrow{i_\varepsilon }(0)\Vert _{2}^2\le \frac{\delta }{\varepsilon ^2}. \end{aligned}$$

As an example, one should think of $\overleftarrow{\rho _{\varepsilon ,\delta }}$ as being equal to $\overleftarrow{i_\varepsilon }(0)$ in the interval $[-\varepsilon +\delta ,-\delta ]$ and smooth in the rest of the support (and similarly for $\overrightarrow{\rho _{\varepsilon ,\delta }}$).

Note that in Theorem 6.2 one can take $L=\varepsilon N$, and this means we can replace the product of occupation variables ${{\bar{\xi }}}^\alpha _x{{\bar{\xi }}}^\beta _{x+1}$ by averages in non-overlapping boxes that correspond to $\langle {{\bar{\pi }}}^{N,\alpha }_s,\overleftarrow{i_\varepsilon }(\tfrac{x}{N})\rangle \langle {{\bar{\pi }}}^{N,\beta }_s,\overrightarrow{i_\varepsilon }(\tfrac{x}{N})\rangle$. Now we want to replace the indicator functions with the functions that we introduced above, i.e. $\overleftarrow{\rho _{\varepsilon ,\delta }}(\cdot -\tfrac{x}{N})$ and $\overrightarrow{\rho _{\varepsilon ,\delta }}(\cdot -\tfrac{x}{N})$ for some $\delta >0$. Under the conditions on $\overleftarrow{\rho _{\epsilon , \delta }}$ and $\overrightarrow{\rho _{\varepsilon , \delta }}$ stated above, we can rephrase the Boltzmann-Gibbs principle as follows.

#### Theorem 6.4

Let $\alpha ,\beta \in \{A,B\}$. For any $0<\delta <\varepsilon$, any $t>0$, and for any function $v\in \ell ^2(\mathbb {T}_N)$6.13

#### Proof

The proof follows from two simple steps. First sum and subtract $\langle {{\bar{\pi }}}^{N,\alpha }_s,\overleftarrow{i_\varepsilon }(\tfrac{x}{N})\rangle \langle {{\bar{\pi }}}^{N,\beta }_s,\overrightarrow{i_\varepsilon }(\tfrac{x}{N})\rangle$, inside the summation in *x* and use the convex inequality $(x+y)^2\le 2x^2+2y^2$. Then, from Theorem 6.2 with $L=\varepsilon N$, we are left to bound the term6.14In order to bound the last display it is enough to control6.15and6.16We present the bound for the first as for the second it is completely analogous. By the Cauchy-Schwarz inequality and Fubini’s theorem, by noting that the random variables correlate in at most $2\epsilon N$ points, we bound (6.15) from above by a constant times

$$\begin{aligned} t\varepsilon N \Big (\sum _{x\in \mathbb {T}_N} v(x)^2\Big ) \int _0^t \mathbb E_{\nu _{\rho }}\Big [\Big (\langle {\bar{\pi }}_s^{N,\alpha },\overleftarrow{i_\epsilon }(0)(\cdot )\rangle \langle {\bar{\pi }}_s^{N,\beta },(\overrightarrow{i_\epsilon }(0)- \overrightarrow{\rho _{\varepsilon ,\delta }})(\cdot )\rangle \Big )^2\Big ]ds, \end{aligned}$$

which, as $\nu _\rho$ is product, can be controlled by a constant times

$$\begin{aligned} t^2\varepsilon \Vert v\Vert _{2,N}^2\Vert \overleftarrow{i_\epsilon }(0)\Vert _{2,N}^2 \Vert \overrightarrow{i_\epsilon }(0)-\overrightarrow{\rho _{\varepsilon ,\delta }}\Vert _{2,N}^2. \end{aligned}$$

To conclude it is enough to use the conditions on the functions $\overrightarrow{i_\epsilon }(0)$ and $\overleftarrow{i_\epsilon }(0)$ as well as (6.12). $\square$

## Mode coupling theory

In this section we give a sketch of the application of the mode coupling theory developed in [36] to our specific model. Note that since the system has two conserved quantities, $\rho ^A$ and $\rho ^B$ (because as we mentioned above $\rho ^C=1-\rho ^A-\rho ^B$), it is possible to have an estimate on the scaling exponent and form of the expected fluctuation limits for quantities *A* and *B*. We refer the interested reader to [29, 36].

Recall (3.8). Then

A.1 $$\begin{aligned} \begin{aligned} {\mathbb {E}}[j^A_{x,x+1}]&=\frac{E_{A}-E_{B}}{N^\gamma }\rho ^{A}\rho ^{B}+\frac{E_{A}-E_{C}}{2N^\gamma }\Big (2\rho ^A(1-\rho ^{A})-2\rho ^{A}\rho ^{B}\Big ),\\ \mathbb E[j^B_{x,x+1}]&=\frac{E_{B}-E_{A}}{N^\gamma }\rho ^{A}\rho ^{B}+\frac{E_{B}-E_{C}}{2N^\gamma }\Big (2\rho ^B(1-\rho ^{B})-2\rho ^{A}\rho ^{B}\Big ). \end{aligned} \end{aligned}$$

From this we get that the current of the system is equal to

A.2 $$\begin{aligned} \begin{aligned} j(\rho )&=\begin{pmatrix} -\nabla \rho ^{A}+\frac{1}{N^\gamma }\Big (\rho ^{A}(1-\rho ^{A})(E_{A}-E_{C})-\rho ^{A}\rho ^{B} (E_{B}-E_{C})\Big )\\ -\nabla \rho ^{B}+\frac{1}{N^\gamma }\Big (\rho ^{B}(1-\rho ^{B})(E_{B}-E_{C})-\rho ^{A}\rho ^{B}(E_{A}-E_{C})\Big ) \end{pmatrix}. \end{aligned} \end{aligned}$$

From last identity, the Jacobian matrix is thus given by

A.3 $$\begin{aligned} J=\frac{1}{N^\gamma }\begin{pmatrix} (1-2\rho ^{A})(E_{A}-E_{C})-\rho ^{B}(E_{B}-E_{C}) &  -\rho ^{A}(E_{B}-E_{C})\\ -\rho ^{B}(E_{A}-E_{C}) &  -\rho ^{A}(E_{A}-E_{C})+(1-2\rho ^{B})(E_{B}-E_{C}) \end{pmatrix}.\nonumber \\ \end{aligned}$$

To obtain the normal modes we need to find the matrix *R* that diagonalizes *J*, $RJR^{-1}=\textrm{diag}(v_\pm )$, where

A.4 $$\begin{aligned} v_{\pm }=\frac{1}{2N^\gamma }[(E_{A}-E_{C})(1-3\rho ^{A})+(E_{B}-E_{C})(1-3\rho ^{B})\pm \delta ], \end{aligned}$$

are the eigenvalues of the matrix *J*. Above

A.5 $$\begin{aligned} \delta =\sqrt{[(E_{A}-E_{C})(1-\rho ^{A})-(E_{B}-E_{C})(1-\rho ^{B})]^2+4(E_{A}-E_{C})(E_{B}-E_{C})\rho ^{A}\rho ^{B}}.\nonumber \\ \end{aligned}$$

The corresponding eigenvectors are given by

A.6 $$\begin{aligned} \tau _{\pm }=\begin{pmatrix} -\frac{1}{2(E_{A}-E_{C})\rho ^{B}}[(E_{A}-E_{C})(1-\rho ^{A})-(E_{B}-E_{C})(1-\rho ^{B})\pm \delta ]\\ 1 \end{pmatrix}.\qquad \end{aligned}$$

Let us now rewrite the previous expressions adapted to the case (I). As observed in Remark 2.4, case (II), $E_B-E_A=E_C-E_A=E\ne 0$, can be deduced by the previous one.

### Case (I): $E_{A}-E_{C}=E_{B}-E_{C}=E$

In this case the expressions above simplify. We get the jacobian matrix

$$\begin{aligned} J=\frac{1}{N^\gamma }\begin{pmatrix}E (1 - 2 \rho _A) - E\rho _{B} &  -E \rho _{A}\\ -E \rho _{B}&  E (1 - 2 \rho _{B}) - E \rho _{A} \end{pmatrix} \end{aligned}$$

and the eigenvalues are

$$\begin{aligned} v_+:=-\frac{1}{N^\gamma }E(\rho _{A}+\rho _{B}-1)\quad \text {and}\quad v_-:=-\frac{1}{N^\gamma }E(2\rho _{A}+2\rho _{B}-1) \end{aligned}$$

and the corresponding eigenvectors are $\tau _+=(-1,1)$ and $\tau _-=(\frac{\rho _{A}}{\rho _{B}},1)$. The matrix $R^{-1}$ and *R* are given by

$$\begin{aligned} R^{-1}=\begin{pmatrix}-1&  \frac{\rho _{A}}{\rho _{B}}\\ 1&  1 \end{pmatrix} \quad \text {and}\quad R=\frac{1}{\rho _{A}+\rho _{B}}\begin{pmatrix}-\rho _{B}&  \rho _{A}\\ {\rho _{B}}&  \rho _{B} \end{pmatrix} \end{aligned}$$

The hessians are

A simple computation shows that  is equal to

and

The coupling matrices $G^1$ and $G^2$ are such that $G^1_{1,1}=0=G^2_{1,1}$, but also $G^2_{2,2}\ne 0$. This means that the first mode is OU and the second is KPZ and this is independent from the choice of the densities $\rho _A, \rho _B$ and $\rho _C$.

We note that the predictions from [36] are done for the strong asymmetric regime, corresponding to the choice $\gamma =0$. But the available techniques still do not allow us to go down the regime $\gamma =1/2$. So what is expected to hold for this model in the KPZ regime is exactly the same behaviour as in the case of a single type of particle i.e. the WASEP, for which one gets for $\gamma >1/2$ diffusive fluctuations and for $\gamma =1/2$ the stochastic Burgers equation. Below the regime $\gamma <1/2$ little is known, but we expect that the same process as for ASEP should appear, i.e. the KPZ-fp.

### The equal density case

Throughout the paper, for sake of simplicity, we have made the assumption $\rho _A=\rho _{B}=1/3$: in this case, the formula (A.5) simplifies and we obtain

$$\begin{aligned} \delta =\frac{2}{3}\sqrt{(E_{A}-E_{C})^2+(E_{B}-E_{C})^2-(E_{A}-E_{C})(E_{B}-E_{C})} \end{aligned}$$

and the eigenvectors (A.6) simplify to

$$\begin{aligned} \tau _{\pm }=\begin{pmatrix} -\frac{c_\pm }{E_{A}{-E_{C}}}\\ 1 \end{pmatrix}, \end{aligned}$$

with eigenvalues

$$\begin{aligned} v_{\pm }=\pm \frac{\delta }{2N^\gamma }. \end{aligned}$$

Above we used the notation in (2.15). To obtain the linear combination of the fields that one should look at, we need to find the matrix *R* that diagonalizes *J*. Observe that $R^{-1}$ is the matrix whose columns are the eigenvectors of *J* so that

$$\begin{aligned} R^{-1}=\begin{pmatrix}-\frac{c_+}{E_{A}{-E_{C}}}& -\frac{c_-}{E_{A}{-E_{C}}} \\ 1&  1 \end{pmatrix} \end{aligned}$$

and its inverse is equal to

$$\begin{aligned} R=-\frac{E_{A}-E_{C}}{3\delta }\begin{pmatrix}1& \frac{c_-}{E_{A}{-E_{C}}}\\ -1& \frac{c_+}{E_{A}{-E_{C}}} \end{pmatrix}. \end{aligned}$$

According to the nonlinear fluctuating hydrodynamic theory, the quantities that we should look at are equal to $R ({{\bar{\xi }}}^A_x, {{\bar{\xi }}}^{B}_x)^T,$ which gives

A.7 $$\begin{aligned} \begin{aligned} \phi _x^+&=-\frac{E_A-E_{C}}{3\delta }{\bar{\xi }}^{A}_x-\frac{c_-}{3\delta }{\bar{\xi }}^{B}_x,\\ \phi _x^-&=\frac{E_A-E_{C}}{3\delta }{\bar{\xi }}^{A}_x+\frac{c_+}{3\delta }{\bar{\xi }}^{B}_x. \end{aligned} \end{aligned}$$

Therefore, the quantities $\phi _x^+$ and $\phi _x^-$ are the conserved quantities that we should look at and on a frame with velocity $v_+$ and $v_-$, respectively. Since we can multiply our fields with suitable constants, we take

The constant *b* is fixed depending on the time scale.

Now that we have the fluctuations fields fixed, let us see the predictions on the form of the fluctuations for each one of these quantities. To do that we look now at the corresponding Hessians of the entries of the jacobian matrix *J*:

A.8

The coupling constants, which are determined by the above matrices, are given on $i\in \{1,2\}$ by  where $R_{i,j}$ is the entry of the matrix *R*. A simple computation shows that

Moreover,

From this we get

$$\begin{aligned} G^1=\begin{pmatrix} 2g_1 &  g_2\\ g_2&  0 \end{pmatrix} \quad \text {and}\quad G^2=\begin{pmatrix} 0 &  g_1\\ g_1 &  2g_2 \end{pmatrix}, \end{aligned}$$

where

$$\begin{aligned} g_1:=-\frac{1}{12N^\gamma }\big \{-c_+^2+(E_{B}-E_{C})(c_+-c_-)+c_-c_+\big \} \\ g_2:=\frac{1}{12N^\gamma }\big \{-c_-^2+(E_{B}-E_{C})(c_--c_+)+c_-c_+\big \}. \end{aligned}$$

Depending on the fact that the constants $g_1$ and $g_2$ are null or not we obtain the limits predicted in Fig. 2, we expect to observe diffusive and/or KPZ behaviour.

In most general case $E_{A}-E_{C}\ne E_{B}-E_{C}$, both modes (A.8) are predicted to have KPZ behaviour. Let us now rewrite the previous expressions in the several particular cases that we have explored above. We start with the case (I): $E_{A}-E_{C}=E_{B}-E_{C}=E$. In this case the expressions above simplify. In the equal density case $\rho _{A} = \rho _{B} = \rho _{C}=\rho =1/3$, we get

A.9 $$\begin{aligned} \delta =\frac{2}{3}E. \end{aligned}$$

The eigenvalues are

A.10 $$\begin{aligned} v_{\pm }=\pm \frac{E}{3N^\gamma }, \end{aligned}$$

with eigenvectors

A.11 $$\begin{aligned} \tau _+=(-1, 1),\quad \tau _-=(1, 1). \end{aligned}$$

The diagonalising matrix *R* and its inverse $R^{-1}$ (the matrix with columns $\tau _+, \tau _-$) are given by

A.12 $$\begin{aligned} R=\frac{1}{2}\begin{pmatrix} -1 &  1\\ 1 &  1 \end{pmatrix},\quad R^{-1}=\begin{pmatrix} -1 &  1\\ 1 &  1 \end{pmatrix}. \end{aligned}$$

Thus, the normal modes are equal to $R({{\bar{\xi }}}^A, {{\bar{\xi }}}^{B})^T$, i.e.

A.13 $$\begin{aligned} \phi _x^+=-\frac{1}{2}{{\bar{\xi }}}^{A}_x+\frac{1}{2} {{\bar{\xi }}}^{B}_x,\quad \phi _x^-=\frac{1}{2}{{\bar{\xi }}}^{A}_x+\frac{1}{2}{{\bar{\xi }}}^{B}_x. \end{aligned}$$

Observe that since we can multiply all the modes by a suitable constant then we take as fluctuation fields the fields in (2.12). Now for the predictions, note that the coupling matrices are equal to

A.14 $$\begin{aligned} G^1=\frac{1}{2}\begin{pmatrix}0&  -\frac{E}{N^\gamma }\\ -\frac{E}{N^\gamma }& 0 \end{pmatrix} \quad \text {and }\quad G^2=\begin{pmatrix}0&  0\\ 0& -\frac{E}{N^\gamma } \end{pmatrix} \end{aligned}$$

which means that the first field should behave diffusively, and the second should behave as KPZ, see [36]. By symmetry the results we obtain in this case also give the same for the case (II): $E_{B}-E_{A}=E_{C}-E_{A}=E\ne 0$. Indeed, in this case, we also have $\delta =\frac{2}{3}E,$ and the eigenvalues of *J* are the same as in case (I), i.e. (A.10), but the eigenvectors are given by $\tau _+=(0, 1)\quad \tau _-=(2, -1).$ Moreover, we have

A.15 $$\begin{aligned} R=\frac{1}{2}\begin{pmatrix} 1 &  2\\ 1&  0 \end{pmatrix},\quad R^{-1}=\begin{pmatrix} 0&  2\\ 1&  -1 \end{pmatrix}. \end{aligned}$$

The normal modes are equal to $R({{\bar{\xi }}}^A_x, {{\bar{\xi }}}^{B}_x)^T$, i.e.

A.16 $$\begin{aligned} \phi _x^+=\frac{1}{2}{{\bar{\xi }}}^{A}_x+{{\bar{\xi }}}^{B}_x,\quad \phi _x^-=\frac{1}{2}{{\bar{\xi }}}^{A}_x. \end{aligned}$$

Since we can multiply by suitable constants we consider then the fields given in (2.13). Similar computations to the ones above, suggest that the first field should behave diffusively, and the second should behave as KPZ.

## A version of Riemann–Lebesgue lemma

In this appendix, we provide a version of the Riemann–Lebesgue lemma for products of stochastic processes for which a bound on the supremum and increments is available. In the context of the present paper, the following proposition is used in Sect. 5 to determine the behaviour of the time-integral of the product of processes living in different time-frames.

### Proposition B.1

Let $\{v_i^N\}_N$, $i=1,\dots , n$, be *n* diverging sequence of constants. Let $T>0$ and, for every $N\in \mathbb {N}$, let  be *n* real–valued stochastic processes on [0, *T*] defined on the same probability space. Assume that there exists $\alpha \in (0,1)$ and $C>0$, such that uniformly in *N*,

B.1

B.2

where, for $s\in [0,T]$, we denoted by .

Then, for any $t\in [0,T]$ and any integers $k_1,\dots ,k_n\in \mathbb {Z}$ such that the limit in (5.1) holds, we have

B.3

where $\lfloor \cdot \rfloor$ denotes the integer part of $\cdot$.

### Proof

Let us introduce the following notation,

B.4

and let  be the integral inside the expectation of (B.3). Let *N* be fixed and, without loss of generality, assume $C_N>0$. Note that

where we used that $e^{-2\pi \iota }=1$, and similarly

By summing up the two previous equalities, we have

B.5

and we will separately bound each of the terms above. Let us begin with $\text {(i)}+\text {(ii)}$. Since by definition (B.4), for all $i=1,\dots ,n$, , by bringing the modulus inside and applying a supremum bound, we have

B.6

where the last step is a consequence of (B.1). For $\text {(iii)}$, we bound the time increment of  between $0\le s_1<s_2\le T$ as

where in the last step we used the definition of  in (B.4). To control the last term, since for any $x\in \mathbb {R}$, $|x-\lfloor x\rfloor |\le 1$, a simple application of Taylor’s formula gives us

$$\begin{aligned} \left| e^{-2\pi \iota \sum _{i=1}^n k_i \frac{\lfloor v_i^N s_j\rfloor -v_i^N s_j}{N}}-1\right| \lesssim \frac{1}{N}\sum _{i=1}^n |k_i|. \end{aligned}$$

Hence, we have

B.7

where in the last step we used (B.2) and (B.1). Since the $k_i$’s are fixed and by assumption $C_N\rightarrow \infty$ as $N\rightarrow \infty$, both (B.6) and (B.7) converge to 0, and the conclusion follows at once. $\square$

## Footnotes

1 Observe that this pairwise balance is not present in the classical *ABC* that we discussed in the first paragraph of Sect. 1.4.

2 This result is stated in [8] but it was not rigorously proved. For a more complicated dynamics with stochastic reservoirs, see the results in [20].
